# High-progesterone environment preserves T cell competency by evading glucocorticoid effects on immune regulation

**DOI:** 10.3389/fimmu.2022.1000728

**Published:** 2022-09-20

**Authors:** Hirofumi Kashiwagi, Toshiro Seki, Shino Oshima, Yusuke Ohno, Tomoka Shimizu, Soga Yamada, Nagi Katano, Yumiko Goto, Atsushi Yasuda, Banri Tsuda, Ryoji Ito, Shun-ichiro Izumi, Hitoshi Ishimoto, Takashi Shiina, Yoshie Kametani

**Affiliations:** ^1^ Department of Obstetrics and Gynecology, Tokai University School of Medicine, Isehara, Japan; ^2^ Department of Internal Medicine, Division of Nephrology, Endocrinology and Metabolism, Tokai University School of Medicine, Isehara, Japan; ^3^ Department of Molecular Life Science, Division of Basic Medical Science, Tokai University School of Medicine, Isehara, Japan; ^4^ Central Institute for Experimental Animals, Kawasaki, Japan; ^5^ Department of Palliative Medicine, Tokai University School of Medicine, Isehara, Japan; ^6^ Institute of Advanced Biosciences, Tokai University, Hiratsuka, Japan

**Keywords:** humanized mouse, pregnant immunity, progesterone, cortisol, specific antibody, T cell, toxic shock syndrome toxin-1, CD62L

## Abstract

Progesterone (P4) and glucocorticoid (GC) play crucial roles in the immunoregulation of a mother to accept and maintain a semi-allogenic fetus. P4 concentration increases during pregnancy and becomes much higher in the placenta than in the other peripheral tissues, wherein the concentration of cortisol (COR), the most abundant GC and a strong immunosuppressor, remains uniform throughout the rest of the body. Here, we evaluated the effect of a high-P4 environment on pregnant immunity by comparing it with COR. Naïve T cell proportion increased transiently in peripheral blood of pregnant women just after delivery and decreased after one month. T cells stimulated with superantigen toxic-shock-syndrome-1 (TSST-1) in the presence of P4 stayed in the naïve state and did not increase, irrespective of the presence of COR, and reactive T cells could not survive. Treatment of T cells with P4 without T cell receptor (TCR) stimulation transiently suppressed T cell activation and proliferation, whereas the levels remain unaltered if P4 was not given before stimulation. Comparison of the engraftment and response against specific antigens using hu-PBL-NOG-hIL-4-Tg mice showed that P4-pretreated lymphocytes preserved CD62L expression and engrafted effectively in the spleen. Moreover, they produced antigen-specific antibodies, whereas COR-pretreated lymphocytes did not. These results suggest that a high-P4 environment suppresses T cell activation and induces T cell migration into lymphoid tissues, where they maintain the ability to produce anti-pathogen antibodies, whereas COR does not preserve T cell function. The mechanism may be pivotal in maintaining non-fetus-specific T cell function in pregnancy.

## Introduction

Immunological tolerance to a semi-allogenic fetus by a pregnant woman is pivotal for the acceptance and maintenance of the fetus and thereby a successful pregnancy. Although pregnant women are at increased risk of ICU admission and ventilation care, the risk of COVID-19-related death is not significantly higher in pregnant women than in non-pregnant women (1, 2). Moreover, the risk of breast cancer progression is not increased in pregnant women compared with that in their non-pregnant counterparts (3). These studies indicate that the immune system is functional in pregnant women as in non-pregnant women. However, whether the mechanism of tolerance to semi-allogenic-grafts can co-exist with a normal defense against cancers or pathogens remains to be clarified.

During pregnancy, the level of gestation-related proteins and hormones, such as progesterone (P4), estrogen, testosterone, and glucocorticoids (GCs), increases. Some of these molecules are correlated with immune suppression (4). Cortisol (COR), the main GC in humans, is regulated by the hypothalamic-pituitary-adrenal (HPA) axis (5), whereas P4, another key steroid hormone in immune regulation during pregnancy, is produced by the trophoblasts independently from the HPA axis in humans. However, the regulatory mechanism of P4 production in placenta has not been fully clarified, although the synthesis pathway has been extensively analyzed (6, 7). In addition, several differences between the functionality of these steroids have been reported (8, 9). By contrast, one study using a mouse model demonstrated that P4 uses GC receptor-dependent pathways for inducing immune suppression and suggested that both steroid hormones have similar effects (10). Although these discrepancies between the previous studies could be attributed in part to the differences in the response of immune cells between humans and mice (11), understanding the orchestrated role of P4 and COR is crucial to understanding the mechanism of immune regulation during pregnancy in humans. Therefore, we hypothesized that establishing a humanized immune system (12, 13) could be useful for analyzing the phenomena caused by the dynamic change of P4 and COR *in vivo*.

P4 is synthesized and secreted by trophoblasts, and the highest concentration of P4 has been reported in the maternal-fetal interface of the human placenta (7), suggesting an important role of P4 in immune regulation during pregnancy. Furthermore, species with a non-invasive placenta, such as mice, do not secrete a large amount of P4 in the placenta (14). However, the placenta of new-world monkeys such as marmosets exhibit the invading characteristics with enhanced P4 production (15, 16). The invasiveness and degree of P4 production are further enhanced in the human placenta (17, 18), which secretes approximately 250 mg of P4 per day (4). Placenta-derived P4 circulates systemically in the blood, and the concentration becomes as high as 20 μM in the villous lumen blood (19, 20). Several studies have explored the role of P4 in immune suppression and unraveled the possible underlying mechanisms (21). For instance, Papapavlou et al. showed that P4 inhibits T cell activation at high concentrations, whereas estradiol does not (22). P4 enhances the expression of human leukocyte antigen (HLA)-G (23), an inhibitory HLA of non-classical type, and progesterone-induced blocking factor (PIBF), an enhancer of calcium influx (14), which play critical roles in immune tolerance and fetal growth. P4 also shifts the T cell function to T cell helper 2 (Th2). Furthermore, P4 blocks Kv1.3, a potassium channel expressed on activated T cells (24). The gene profile of P4-regulated immune cells has also been clarified (25). However, the precise mechanism of how P4 regulates T cell activation is elusive.

COR produced mainly in the adrenal gland is pivotal for early pregnancy (26). COR is a multi-functional transcription factor regulator and strongly suppresses the production of inflammatory cytokines secreted by innate immune cells (27, 28). Moreover, COR modulates and suppresses T cell activation directly and indirectly by inducing Treg cells and Th2 cells (27, 28). Therefore, COR or its derivatives are used in clinical cases as strong immune suppressors. In contrast to P4, COR is regulated *via* the HPA axis during pregnancy (29, 30), and its level in the peripheral blood is comparable with that of P4 in the second to third trimesters. In addition, COR enhances the immune reaction by controlling the distribution and response of T cells (31). Although COR is produced in the mother’s adrenal gland, it is blocked in the syncytiotrophoblast because of the presence of 11-b-hydroxysteroid dehydrogenase, which reacts with COR to produce non-reactive cortisone (32). In contrast, the level of COR increases under stress conditions, allowing it pass the syncytiotrophoblast, making an immune-suppressive signal to the fetus (33). This phenomenon suggests a crosstalk pattern in the mother and fetus endocrine system.

Hence, considering the difference in steroidogenesis regulation, we speculated that the kinetics might be closely related to the bi-directional immune regulation of tolerance to semi-allograft fetus and defense against cancers and/or pathogens during pregnancy. Therefore, we aimed to comprehensively compare the effects of P4 and COR on human lymphocytes *in vitro* and *in vivo* to clarify their role in the systemic regulation of immunity during pregnancy.

To stimulate T cells, we used superantigen TSST-1, which can stimulate large numbers of T cell repertoires in the presence of antigen presenting cells (APCs) (34). Superantigens induce transient T cell activation by T cell receptor (TCR)-major histocompatibility complex (MHC) crosslinking, following anergy induction and regulatory T cell differentiation (35, 36). However, superantigens induce a state of steroid resistance in activated T cells by non-canonical pathway (37). Because we aimed to compare the APC-involved T cell response in the presence of steroids, we selected one of such superantigens, TSST-1 for *in vitro* stimulation of T cells.

Because the functional assay of specific antibody production is very difficult *in vitro*, and the mouse immune system is different from that of humans, we used humanized mouse system. Humanized mouse system with human immunity has been investigated extensively. The human immune system was developed by transplanting human tissues such as hematopoietic stem cells (HSCs) or PBMCs into immunodeficient mouse (38–40). There mice could engraft human immune cells and immune reactions could be monitored by the system. Among them, the hu-PBL-hIL-4-Tg-NOG mouse system, which prevents graft versus host disease (GvHD) in the xeno-PBMC transplantation, induces antigen-specific antibody production by human immune cells *in vivo* (41, 42). The mouse system develops the morphology of peripheral lymphoid tissues similar to that of the spleen and lymph node, constructing white and red pulp (personal communication). Therefore, compared with other humanized mouse systems, the hu-PBL-hIL-4-Tg-NOG system can present a B cell response more comparable to that of humans. Using the mouse model, we identified the difference between the effects of the two steroids on the engrafted human PBMCs *in vivo*.

## Materials and methods

### Ethical approval

Human PBMCs were derived from healthy donors or pregnant women after receiving written informed consent following protocol approval by the Institutional Review Board according to the institutional guidelines. This work was approved by the Tokai University Human Research Committee (approval no. 20R211, 21R277) and the Central Institute for Experimental Animals (CIEA) Human Research Committee (08–01). The experiments were conducted in accordance with the guidelines of the Declaration of Helsinki and the Japanese Federal Regulations required for the protection of human subjects. Immunodeficient mice were used for xenotransplantation studies in compliance with the Guidelines for the Care and Use of Laboratory Animals, and all animal protocols were approved by the committees of CIEA (#20045) and the Tokai University School of Medicine (#185016, #191073, #202049, and #213047). This study was carried out in compliance with the ARRIVE guidelines.

### Clinical samples

Peripheral and placental blood were obtained from healthy donors or pregnant women without a history of malignant diseases. A total of 56 healthy donors without pregnancy (age, 21–61; 37 males and 19 females) and 15 pregnant women (age, 23–42) were enrolled in this study. The list of pregnant women is shown in **Table S1**.

We used NOG-hIL-4-Tg (formal name, NOD.Cg-Prkdc*^scid^* Il2rg*^tm1Sug^*/Tg(CMV-IL4)/Jic) mice and transplanted PBMCs obtained from healthy donors that were treated with P4 or COR into them. To examine the effector function of these treated PBMCs, the function of T and B cells could be evaluated because human PBMC can engraft in the mouse without causing GvcHD (41, 42). The NOG-hIL-4-Tg mice were housed under specific pathogen-free conditions in the animal facility located at CIEA or Tokai University School of Medicine during the experiments. DNA was extracted from the ear tissues collected at the time of genotyping. Offspring with the expression of human IL-4 were identified as described previously (41).

### Preparation of human PBMCs

Approximately 30 mL blood from healthy donors was drawn into Vacutainer ACD tubes (Becton Dickinson, NJ, USA) containing heparin. The collected sample was immediately placed in 10 mL of Ficoll-paque PLUS (Cytiva, UK, London), and mononuclear cells were isolated by density centrifugation (500 × *g*, 30 min, 20°C). The cells were washed with phosphate-buffered saline (PBS) for 5 min at 300 × *g*, 4°C, and used for the *in vitro* and *in vivo* analyses.

### *In vitro* culture of PBMCs

PBMCs (1×10^6^/mL) was stimulated with 1 μg/mL toxic shock syndrome toxin-1 (TSST-1) (Toxin Technology Inc, Sarasota, USA) in RPMI-1640 medium (Nissui Co. Ltd, Tokyo, Japan) supplemented with 10% Fetal Calf Serum (FCS) (Sigma-Aldrich, St. Louis, USA) and antibiotics (streptomycin, 0.1 mg/mL, penicillin 100 unit/mL; Meiji Seika, Tokyo, Japan) in the presence or absence of different amounts of progesterone (P4) (Sigma-Aldrich) and/or cortisol (COR) (Sigma-Aldrich) at 37°C for 72 h with 5% CO_2_. Cells were collected, washed with PBS, and 5×10^5^ cells/tube were submitted for flow cytometry (FCM) analysis.

For the re-stimulation analyses, 7×10^6^ PBMC/mL were cultured in RPMI-1640 with 10% FCS and the antibiotics, as mentioned above, in the presence of 0–200 μM COR or P4 37°C for 6 h with 5% CO_2_. Cells were collected, washed with PBS, and re-cultured in various conditions. After the treatment, cells were collected and submitted for the FCM analysis.

Three independent experiments were conducted as follows. Exp. 1; PBMC was cultured for 6 h in the presence of 200 μM P4 or COR, washed, and stimulated by TSST-1 in the presence or absence of the same steroids. For negative and positive controls, cells with no stimulation and cells with TSST-1 stimulation were used, respectively. Exp. 2; T cells were stimulated with TSST-1 for 6 h, and after the stimulation, P4 or COR was added to the cells. Exp. 3; T cells were stimulated with TSST-1, and after the stimulation, TSST-1 was depleted, and P4 or COR was added to the cells. After 48 h, T cells were re-stimulated with TSST-1,

### Cell division of T cells

T cells were purified using Pan T cell Isolation Kit, human (Miltenyi Biotec, Bergisch Gladbach, Germany). Briefly, PBMCs were cultured in the presence or absence of TSST-1 and P4 or COR for 72 h. Subsequently, the cells were collected, washed, and incubated with the Pan T cell biotin-antibody cocktail at 4 °C for 5 min. After adding 40 µL wash buffer, the Pan T cell microbead cocktail (20 µL) was added and incubated at 4 °C for 10 min. The T cells were sorted using the Automacs system (program: depletion; Miltenyi Biotec) and labeled using the CellTrace™ Cell proliferation kit (Thermo Fisher Scientific, MA, USA) following the manufacturer’s instructions. 5- (6)-Carboxyfluorescein diacetate succinimidyl ester (CFSE) powder was reconstituted with dimethyl sulfoxide (Wako, Tokyo, Japan) at a concentration of 5 mM. The cells were incubated with CFSE (final concentration, 5 µM) at 37 °C for 20 min in the dark, washed with PBS, and resuspended in RPMI-1640 medium. Afterward, the cells were stimulated with Dynabeads™ Human T-Activator CD3/CD28 (Thermo Fisher Scientific) or an anti-CD3 coated microplate. Cells were collected, and the cell cycle was analyzed using FCM for 3 days, as described below.

### FCM analysis

Fluorochrome-conjugated anti-human monoclonal antibodies (mAbs) were used to identify human immune cells. The cells were incubated with appropriate dilutions of fluorescently labeled mAbs for 15 min at 4°C and were then washed with PBS containing 1% (w/v) Bovine Serum Albumin (BSA) Sigma-Aldrich, St. Louis, USA). The cells were analyzed using FACS Fortessa, or Verse (BD Bioscience, Franklin Lakes, NJ, USA). For each analysis, the live gate with white blood cells or lymphocytes was further gated based on hCD45 expression. The data were analyzed using FlowJo™ v10 (BD Bioscience). The mouse anti-human mAbs used in this study are shown in **Table S2**.

Culture supernatants were collected, and the debris was removed by centrifugation at 300xg at 4°C. Cytokine quantitation was conducted using LEGENDplex (BioLegend) according to the manufacturer’s instructions. Briefly, 25 µL of the supernatant was mixed with 25 µL capture beads and incubated for 2 h at 25°C. The beads were washed, mixed with detection antibodies, and incubated for 1 h at RT. Subsequently, streptavidin-phycoerythrin was added, and the mixture was incubated for 30 min at 25°C. Finally, the beads were washed and analyzed using FCM. Analysis was performed using the BD FACSVerse™ Flow Cytometer (BD Biosciences, Franklin Lakes, NJ, USA). The data were analyzed in pg/mL using LEGENDPlex™ V8.0 (BioLegend, San Diego, USA).

### Transplantation and treatment of PBMC-NOG-hIL-4-Tg mice

PBMCs (1×10^6^/mL) were cultured in RPMI-1640 with 10% FCS equipped as mentioned above in the presence or absence of 200 μM COR or P4 at 37°C for 6 h with 5% CO_2_. Cells were collected, washed with PBS, and 5 x10^6^ PBMC were transplanted intravenously into 8- to 9-week-old NOG-hIL-4-Tg mice.

A CH401 peptide, which includes the epitope sequence of the anti-HER2 mAb, was determined using MAP-peptides with a partial amino acid sequence of HER2/neu (N:YQDTILWKDIFHKNNQLALT) (43). The peptide was synthesized using a Rink amide resin (0.4–0.7 mmol/g), an ACT357 peptide synthesizer (Eurofins Genetics Tokyo JPN), and a multiple antigen peptide named CH401MAP, a 20-mer peptide of the HER2 molecule that was synthesized as an antigen peptide. HER2 peptide was emulsified with complete Freund’s adjuvant (CDA) (Becton Dickinson) (50 μg/head, 100 μL 1:1/v:v) and administered to the PBMC-NOG-hIL-4-Tg mice intraperitoneally. For the negative control, an equal volume of PBS was emulsified and injected into PBMC-NOG-hIL-4-Tg mice transplanted with the same PBMCs obtained from healthy donors. Boosters were administered using incomplete Freund’s adjuvant (IFA) (Wako Pure Chemical Industries, Ltd) 2 weeks after the first immunization. At 4 weeks after transplantation and 2 weeks after booster administration, peripheral blood (PB) was collected in the presence of heparin *via* retro-orbital bleeding under inhalation anesthesia. The mice were sacrificed and analyzed for T and B cell profiles using FCM after 28 days. Antibody production was analyzed using hybridoma preparation and ELISA, as described below.

### Hybridoma preparation of human B cells engrafted in the spleen of the hu-PBL hIL-4 NOG mouse

The hu-PBL hIL-4 NOG mice with >1×10^7^ spleen cell numbers were selected. The mouse splenocytes were fused with the mouse myeloma cell line, P3-X63-Ag8-U1, using electroporation with a BEX CFB16-HB (BEX co. LTD, Tokyo, JPN) and the electrode, LF497P2 as reported previously (42). The conditions for electroporation were set at AC 30 V for 20 s, DC 350 V for 30 µs/500 ms DC cycle 3, AC 30 V for 7 s, Fade on, in electrofusion buffer (0.3 M mannitol, 0.1 mM calcium chloride, and 0.1 mM magnesium chloride). Fused cells were cultured under the condition (1×10^7^/mL). The cells were cultured in hypoxanthine, aminopterin, and thymidine-containing (HAT) medium for two weeks. ELISA was conducted using the supernatants to measure total Ig, CH401MAP-specific IgG, and BAM6MAP-specific IgG, as described previously.

### Enzyme-linked immunoassay (ELISA)

The level of human IL-4 protein was measured using the Human IL-4 ELISA Set BD OptEIA™ (BD Biosciences) according to the manufacturer’s instructions. The protocol for specific IgG antibody detection has been previously described (41). Briefly, micro-wells of microtiter plates (Thermo Fisher Scientific) were coated with anti-human Igs (for total IgG detection), CH401MAP (for specific IgG detection), or BAL6MAP, a third-party antigen (N:NYYAGGFNSVRGFKDSTLGP) (1 μg/mL) diluted in carbonate buffer (pH 9.5), and the antigens were adsorbed to microtiter plates overnight at 4°C. The wells were washed with PBS-Tween (0.05% v/v) and blocked with 3% BSA-PBS at RT for 2 h. After three washes with PBS-Tween, 10-fold serial dilutions of mouse plasma were added to the wells and incubated for 2 h at RT. The plates were washed three times before adding biotin-conjugated mouse anti-human IgG mAb (BD Pharmingen, San Diego, USA) (1:3,000). After 2 h incubation at 37°C, the plates were washed 3 times, followed by the addition of streptavidin-horseradish peroxidase (1:50,000 v/v; BD Pharmingen). The plates were incubated for 1 h at RT, and unbound conjugates were removed by washing. Then, the EIA substrate kit solution (Bio-Rad Laboratories, Hercules, CA, USA) was added to each well. The reaction was stopped with 10% HCl, and the absorbance was measured at 450 nm.

### Immunohistochemistry

NOG-IL-4-Tg mouse spleen and lung tissues were fixed with Mildform^®^ (FUJIFILM Wako Pure Chemical co. Osaka, Japan) and embedded in paraffin. A paraffin block was micro-sectioned and de-paraffinized. The tissue sections were mounted onto glass slides after fixation using formalin, washed, and endogenous peroxidase was blocked for 10 min at RT. The sections were blocked with goat serum for 30 min, washed, and then the primary monoclonal antibody was added. Subsequent incubation with peroxidase-labeled anti-mouse Ig was performed according to the manufacturer’s protocol. The antibodies are shown in **Table S4**.

### Statistical analysis

All statistical analyses were performed using Microsoft Excel (Microsoft, Redmond, WA). The data are shown as the mean ± standard deviation (SD). Significant differences between groups were determined using one-way ANOVA or two-sided Student’s *t*-test analysis.

## Results

### Reduced P4 induces naïve T cell mobilization in the blood flow after the delivery

**Figure 1:**
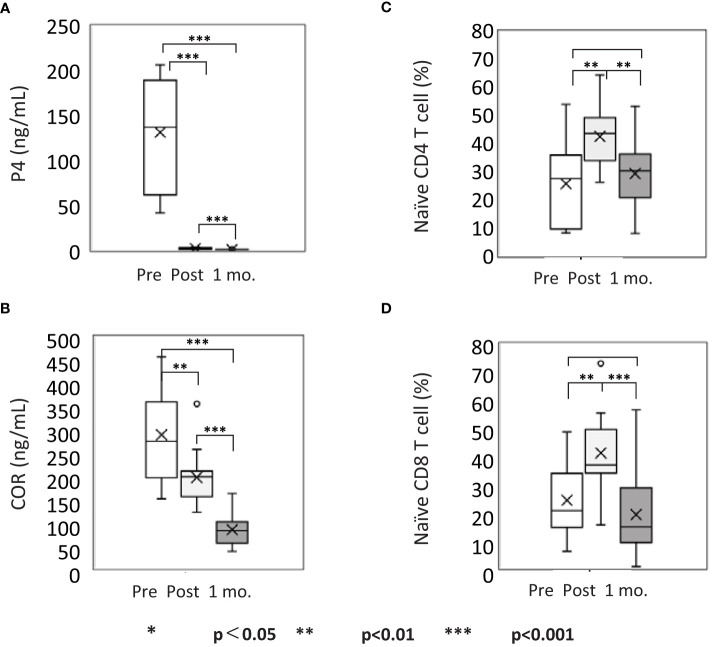
Naïve T cell mobilization coincides with progesterone (P4) decrease in the peripheral blood after delivery. The kinetics of steroids and T cells in the PBMCs of pregnant women. Pre- and post-delivery concentration of **(A)** P4 in the plasma (ng/mL); **(B)** cortisol (COR) in the plasma (μg/mL); **(C)** naïve CD4 T cell (%) in CD4^+^ cell gate; **(D)** naïve CD8 T cell (%) in CD8^+^ cell gate. pre, pre-delivery; post, post-delivery; 1mo, one month after delivery. *p<0.05; **p<0.01; ***p<0.001 [one-way analysis of variance (ANOVA)]; n = 15. See also **Table S1** and **Figure S1**.

P4 and COR are representative steroid hormones that regulate the immune system. Therefore, we measured their concentrations in the plasma 0–3 days before (hereafter referred to as before), 3–4 days after (hereafter referred to as after), and 27–39 days after (hereafter referred to as after one month) delivery (**Table S1**). The mean serum concentration of P4 was 131.22 ng/mL before and 1.25 ng/mL after delivery, which decreased to 0.27 ng/mL after one month (**Figure 1A**). The mean COR significantly decreased from 288.4 ng/mL to 195.8 ng/mL and finally to 82.7 ng/mL, respectively; however, the range of reduction was lower than that of P4 (**Figure 1B**). Furthermore, to understand the relation between these changes in P4 and COR with T cells, we analyzed the kinetics of T cells in the peripheral blood before and after the delivery. The mean proportion of naïve CD4 T cells significantly increased from 25.62% (before) to 42.55% (after) and significantly decreased to 29.24% (after one month) (**Figures 1C** and **S1A**), whereas that of naïve CD8 T cells increased from 24.08% to 40.96% and decreased to 18.96% (**Figures 1D** and **S1A**), respectively. These differences were significant, suggesting that naïve T cells transiently increase in the PB of a mother and return to the baseline level one month after the delivery. The prominent decrease in P4 and COR levels indicated that environmental change may affect the kinetics of naïve T cells. Because P4 enhances the expression of CD62L, a marker for lymph node migration (44), it might play a pivotal role in preserving naïve T cells in lymph nodes during pregnancy. Therefore, we further analyzed the effects of P4 and COR on the naïve T cell kinetics and localization in the lymphoid tissues.

### T cell activation and proliferation are suppressed dominantly by P4 to GC

A P4 concentration of between 20 and 200 μM, i.e. similar to the concentration in the intervillous blood of the placenta of third trimester, rapidly decreases the gene expression related to nuclear factor of activated T-cells of T cells (20, 21, 24). The highest concentration of COR in circadian variation or in the third trimester intervillous blood is nearly 20 μM. Therefore, we selected 20 and 200 μM (10 X of 20 μM) and assessed the changes in the expression of activation markers on the lymphocytes after T cell stimulation using FCM. As superantigen stimulation induces T cell activation in the presence of GC (37, 45), we stimulated PBMCs with TSST-1 in the presence of P4 and/or COR and analyzed the expression of CD25 and PD-1 after 72 h.

**Figure 2:**
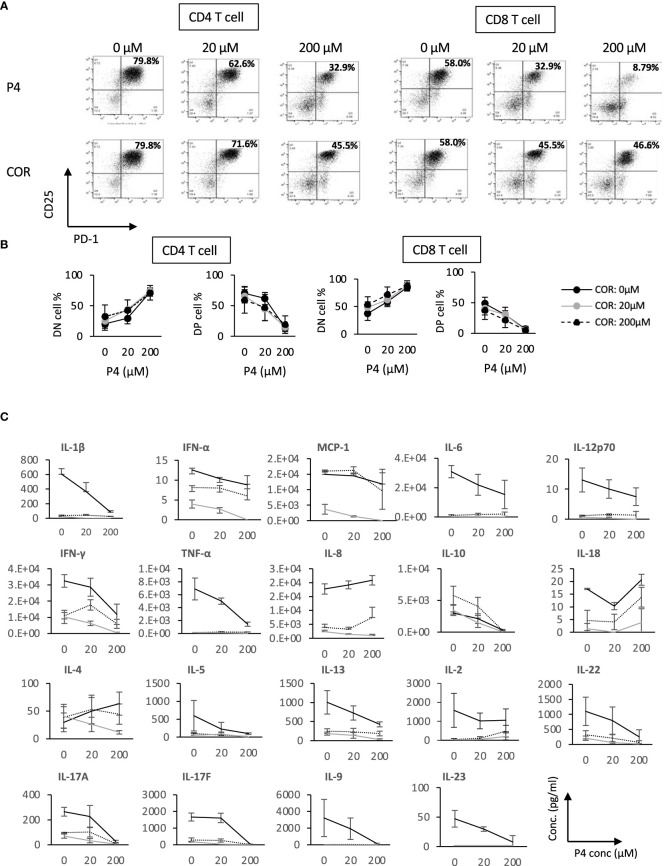
P4 and COR effects on T cell activation *in vitro*. **(A)** The expression patterns of activation markers (CD25 and PD-1) on T cells after TSST-1 stimulation cultured for 72 h in the presence of 0–200 μM P4 or COR. Upper panels; P4 treatment. P4 concentration is shown above each panel. Lower panels; COR concentration is shown above each panel. Left four panels; CD4 T cells, Right four panels; CD8T cells. **(B)** Kinetics of CD25/PD-1 double negative (DN; non-activated) and double-positive (DP; fully activated) T cell ratios. Upper panels; P4 concentration is shown on the horizontal axis. Lower panels; COR concentration is shown on the horizontal axis. Left four panels; CD4 T cells, Right four panels; CD8T cells. **(C)** Concentration of inflammatory and T cell subset-related cytokines in the culture supernatants. X-axes show the concentration of P4 in the culture medium, and Y-axes show the concentration of inflammatory and T cell subset-related cytokines. Solid lines, no COR; dotted lines, 20 μM COR; gray lines, 200 μM COR; Data show average ± standard deviation (SD); n = 3. See also **Figures S1** **S2**.

The proportion of CD25/PD-1 double-positive (DP) cells decreased with an increased P4 concentration, while that of double-negative (DN) cells increased in both CD4 and CD8 T cells. On the contrary, no significant changes in both DP and DN cells were observed with changes in COR concentration (**Figure 2A**). The results also demonstrated that in the presence of P4, the proportion of activated T cells with expression of the activation markers was significantly decreased irrespective of COR concentration. In other words, no significant effects of COR were observed in the suppression of T cell activation. Both CD4 and CD8 T cells showed similar results (**Figure 2B**). Moreover, the number of DN T cells was not changed with P4 addition, suggesting that P4 did not increase the DN cell number but suppressed DP differentiation (**Figure S2**).

The proportion of CD45RA^−^CD62L^−^ effector T cells (Teff) decreased in a P4 concentration-dependent manner. By contrast, the proportion of CD45RA^+^CD62L^+^ cells tended to be maintained. However, the surface expression of CD45RO and CD95, the memory and effector T cell markers, was not increased in the presence of P4, suggesting that the T cells, which are arrested in a non-activated state, maintain naïve cell characters (**Figure S3**). Some difference from normal naïve T cells was observed, as CD127 expression was increased by COR and CD197 expression was decreased by P4 (**Figure S3**).

Next, we examined the level of inflammatory cytokines secreted by these PBMCs (**Figure 2C**). The concentration of most of the cytokines was lower in the COR-treated supernatants than that in the P4-treated supernatants. However, IL-17F secretion was lower in the P4-treated supernatants (200 μM; 40.80 + 23.57 pg/ml) compared to that in COR-treated supernatants (200 μM; 181.79 + 27.36 pg/ml, p<0.01). IL-10 was increased in the presence of 20 μM COR while P4 suppressed IL-10 secretion in a concentration-dependent manner. Cytokines secreted by non-T cells, such as IL-12p70, IFN-α, MCP-1, IL-8, and IL-18, were significantly suppressed by COR-treatment, whereas the effects of P4 were not significant. The addition of P4 and COR (at 200 μM) completely suppressed almost all of the cytokines examined, except for IL-18 and IL-2. Furthermore, IL-4 was increased by P4 addition, but it was suppressed in the presence of COR. These results indicate the potential efficiency of P4 in suppressing the inflammatory cytokines, which is further enhanced by the presence of COR.

**Figure 3:**
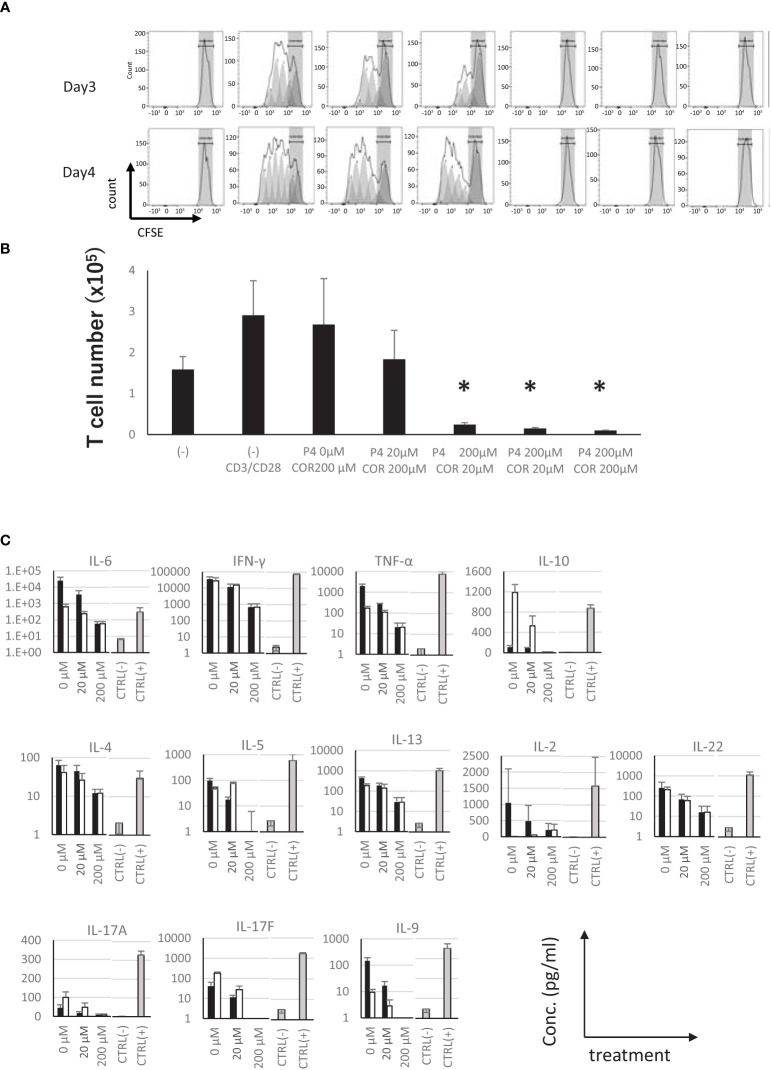
Effects of presence of P4 and COR on the proliferation of T cells. **(A)** 5-(6)-Carboxyfluorescein diacetate succinimidyl ester (CFSE) attenuation on the purified T cells 3 (upper panels) and 4 (lower panels) days after stimulation in the presence of P4 and/or COR. Gray zones represent the ranges of non-dividing cells. Gray peaks are dividing cells predicted from the raw data. Typical patterns of 3 independent experiments are shown. **(B)** T cell number 3 days after the proliferation was shown. *p < 0.05 (Student’s t-test). **(C)** Concentration of T cell subset-related cytokines in the culture supernatants. Horizontal lines show the concentration of P4 or COR in the culture medium. CTRL (−); negative control, culture supernatants without stimulation by TSST-1. Gray bars, CTRL (+); positive control, culture supernatants stimulated only by TSST-1; Closed bars, 200 μM P4 was added; Open bars, 200 μM of COR was added; Each data are mean ± SD; n = 3.

Furthermore, to examine whether these steroids directly affect the T cells, we used purified and CFSE-labeled T cells to examine the effects on proliferation. The results showed that the proliferation of T cells was completely suppressed in the presence of 200 μM P4, while no inhibition was observed in the presence of COR up to 200 μM (**Figure 3A**). Moreover, almost all the cytokines produced by T cells were decreased in the presence of P4 dose-dependently. COR showed no effects on proliferation of T cells. The production of cytokines was completely suppressed in the presence of both steroids, and approximately 80% of cells were killed by the treatment (**Figures 3B, C**).

Together, these results suggest that P4 suppresses T cell activation, proliferation, and cytokine production in a concentration-dependent manner, maintaining the naïve phenotype, but induces cell death in purified T cells. The suppression by P4 was dominant to the proliferative effect of COR, suggesting that the T cell fate is mainly regulated by P4 in high-P4 and COR environment.

### P4 transiently suppresses the activation of T cells after T cell receptor (TCR) stimulation

**Figure 4:**
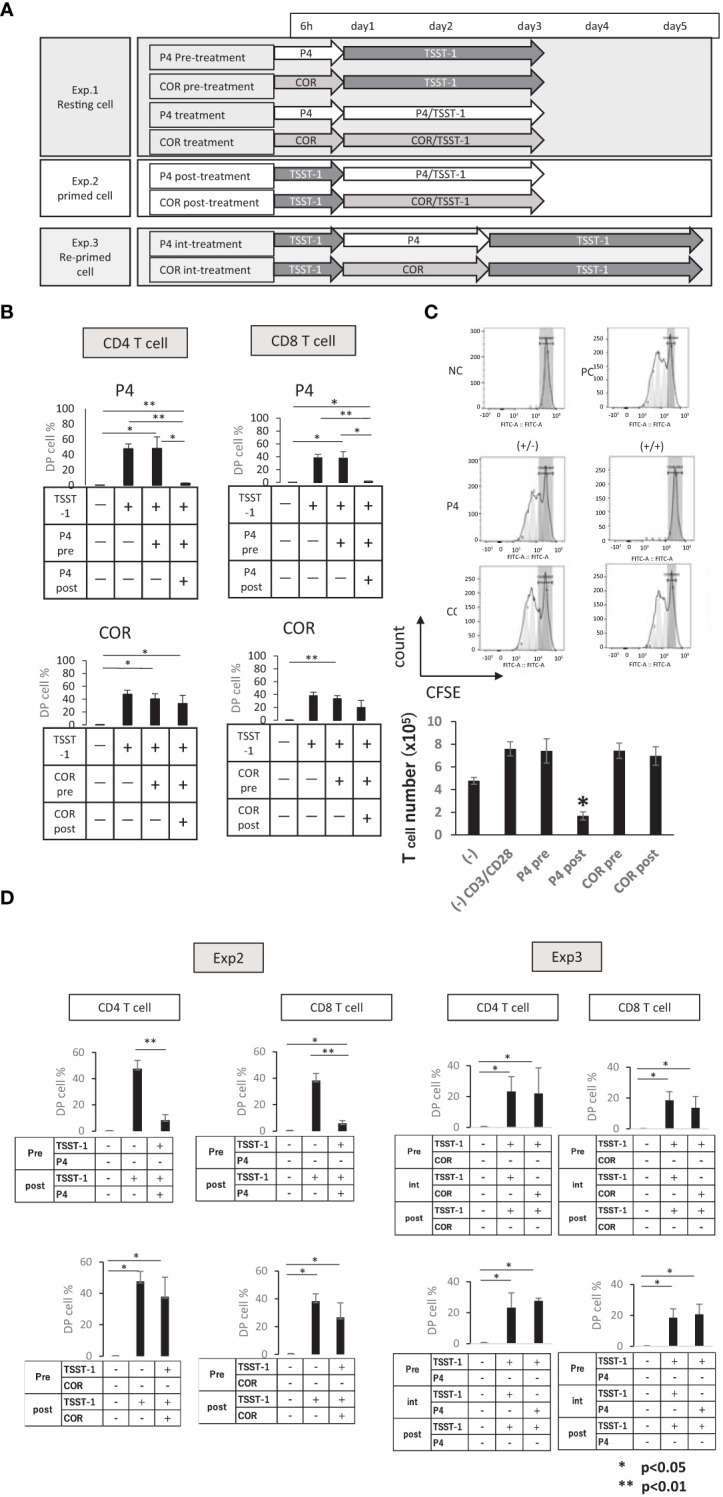
Effects of P4/COR pre/post-treatments on T cell activation. The PBMCs were treated with/without P4 or COR before stimulation by TSST-1. **(A)** The experimental design of pre/post-treatment with P4/COR. Three experiments were designed as shown. **(B)** Experiment 1 (Exp. 1)— CD25/PD-1 DP cell ratios (%) in CD3 T cells are shown. The treatments are shown in each table below the panel. The solid bars show mean ± SD; n = 3. *p < 0.05 (Student’s t-test) **(C)** (Exp. 1) CFSE attenuation on the purified T cells 3 days after stimulation. Upper panels: NC, negative control; PC, positive control; (+/-), pretreatment groups; (+/+), treatment groups. Gray zones represent the ranges of non-dividing cells. Gray peaks are dividing cells predicted from the raw data. Typical patterns of three independent experiments are shown. Lower graph: (−), negative control; (−) CD3/CD28, positive control; P4 pre, P4 pre-treatment group; P4 post, P4 treatment group; COR pre, COR pre-treatment group; COR post, COR treatment group. **(D)** (Exp. 2 (left panels) and Exp. 3 (right panels)). CD25/PD-1 DP cell ratios (%) in CD3 T cells are shown. The treatments are shown in each table below the panel. The solid bars show mean ± SD; n = 3. *p < 0.05, **p < 0.01 (One-way ANOVA and Student’s t-test).

When resting and/or primed T cells are exposed to a high concentration of P4 in the placenta, they may stay there for a short time and be left there if it is not activated by an alloreactive antigen. After the experience of a high P4 environment, T cells might change the activation status to react to a third-party antigen. Therefore, we tried to examine if a high concentration of P4 influences the level of activation on T cells. Three experiments were designed to examine the effect of P4 (**Figure 4A**). In Exp. 1, resting cells were pre-treated with P4 or COR for 6 h before TSST-1 stimulation. The P4 pretreatment group was activated at a similar level as the positive control, while no activation was observed in the P4 treatment group (**Figure 4B**). By contrast, the activation of the cells in the COR pretreatment group was not suppressed significantly. Similar phenomena were observed in CD4 T cells and CD8 T cells, with no significant difference. These results suggest that TCR stimulation in the presence of P4 strictly suppresses T cell activation, but T cells can be activated by P4 pretreatment. COR treatment slightly decreases the activation after TCR stimulation, but does not strictly affect the T cell activation.

Next, we analyzed the proliferation of T cells after the pre-treatment. The P4 pretreatment group proliferated, while the P4 treatment group induced cell death, and no proliferation was observed (**Figure 4C**). In addition, all cytokines except IL-4 tended to be suppressed or maintained at the same level; IL-4 increased significantly in the P4 treatment group (**Figure S4**). These results confirmed that pre-treatment by P4 without TCR stimulation did not affect T cell proliferation and activation. By contrast, the longer period of TCR stimulation under a high P4 environment was deleterious for T cells.

Finally, we designed two further experiments, to clarify if P4 suppression of T cells is superior to preceding activation signal (Exp. 2) or transient/permanent (Exp. 3). The results showed that P4 suppressed TCR-pre-stimulated T cell activation significantly. However, the re-stimulation without P4 induced T cell activation at the same level as that of the positive control (**Figure 4D**), suggesting that P4 suppression is transient. Contrary to this, COR showed no significant effect on the activation. Moreover, Exp. 1 and Exp. 3 suggested that P4 treatment without TCR stimulation is not toxic to human T cells because the response following P4 treatment is comparable to the response in the positive control.

### Progesterone maintains the naïve phenotype of T cells after TCR stimulation

Next, we analyzed the surface markers of T cells in detail to clarify the differentiation level of these T cells. The P4-pretreatment group showed that the proportion of CD45RA^+^CD62L^+^ cells was comparable to the positive control (TSST-1 stimulation only) (**Figure S5A**). The mean fluorescent intensity (MFI) of CD95 in these DP cells was also increased to the same level as the positive control, suggesting a significant part of cells committed to memory cells. Further marker analyses revealed that CD127, CD197, CXCR3, and CD45RO markers showed a similar pattern to the positive control, suggesting the significant effect was not induced by the P4 pretreatment (**Figure S5B**). By contrast, the P4 treatment group decreased the live cell number, and the expression of the differentiation markers was similar to that of the non-stimulated negative control. The proportion of CD45RA^+^CD62L^+^ cells was also higher in the P4 pre-treatment group than in the P4 post-treatment group, and the expression of CD95 was low, suggesting that the cells were maintained in the naïve state in the presence of P4.

These results of surface marker expression suggest that P4 arrests T cell activation and memory-cell differentiation into stem cell-like memory T cells (TSCM; defined as CD45RA^+^CD62L^+^ CD45RO^+/-^CD197^+^CD127^+^CD95 ^±^ CXCR3^+^ cell), the earliest stage of memory T cells development. To confirm these results, we examined the expression of FOXM, a member of the Forkhead Box (Fox) family of transcription factors involved in the proliferation and differentiation of functional effector T cells, which is a marker of differentiation into TSCM cells by Notch signal (46). In the P4 treatment (pre + post) group, the expression of FOXM1 was lower compared to that in the P4 pre-treatment and COR treatment (pre + post) group, suggesting that P4 tends to suppress the differentiation of naïve T cells into TSCM (**Figure S6**).

### P4-treated PBMC can engraft and maintain CD62L expression

**Figure 5:**
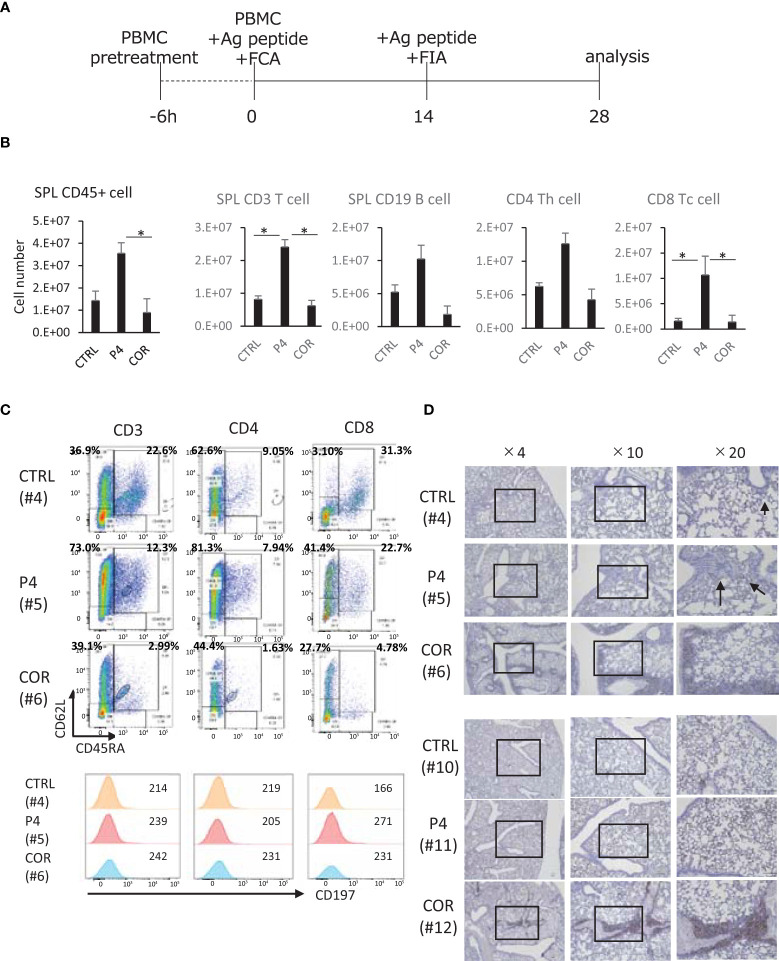
Effects of P4/COR pre-treatment on humanized immune reconstitution in humanized mice. The NOG-hIL-4-Tg mice were used for the humanized mice carrying immune conditions. **(A)** Protocol for the animal treatment. **(B)** Analysis of human lymphocytes engrafted in the mouse spleens. Spleen cells were prepared after 28 days, and the cell number was counted. FCM was conducted, and human CD45^+^ leukocytes, CD3^+^ CD4^+^, CD8^+^ and CD19^+^ cells were detected by monoclonal antibodies. From the ratio and spleen cell number, the total cell number of each subset was calculated and shown. The solid bars show mean ± SD; n = 4; *p<0.05 (Student’s t-test). **(C)** Typical patterns of spleen T cells are shown. CTRL (upper panels), the profile of control mouse spleen cells; P4 (middle panels), the profile of P4 mouse spleen cells derived from P4-treated PBMCs engrafted in the spleen; COR (lower panels), the profile of COR mouse spleen cells derived from COR-treated PBMCs engrafted in the spleen. Cells were gated for lymphocyte (FSC and SSC), CD3, CD4, and CD8. **(D)** Typical immunohistochemical patterns of lung tissue sections stained with anti-human CD45 (Mouse #4, #5, #6, #10, #11, and #12) are shown in **Table S3**. Two sets of healthy mice donors were selected, because CD45^+^ cell % of COR-treated mouse spleens were sufficiently high. This suggests that the engraftment is comparable to other treatments of the Two sets of healthy mice donors were selected because CD45^+^ cell % of COR-treated mouse spleens were sufficiently high. This suggests that the engraftment is comparable to other treatments of the sets of mice. Open squares show the sites of enlarged images. See also **Figure S7**.

Next, we transplanted healthy donor PBMCs treated with 200 μM P4 or COR for 6 h into these mice and immunized them with CH401MAP, an antigen peptide of HER2, biweekly (**Figure 5A**). We used PBMCs of non-pregnant healthy donors because the T cells had not been exposed to a high level of P4 or COR. The results revealed a significantly higher number of human CD45^+^ cells engrafted in the spleens of P4- than in COR-treated mice 4 weeks after the transplantation (**Figure 5B**). The engrafted human CD45^+^ cells contained T and B cells, and the CD8^+^ T cells were significantly higher in P4-treated mice compared to both control and COR-treated mice. Compared with control and P4-treated mice, COR-treated mice tended to engraft fewer human CD45^+^ cells. B cell engraftment tended to be lower in COR-treated mice than in control mice.

Furthermore, flow cytometry (FCM) analyses for assessment of CD45RA and CD62L expression revealed that in P4 and COR treatments, the proportion of CD45RA^+^CD62L^+^ cells tended to decrease. The tendency was prominent in the COR-treated mice, while P4-treated mice preserved more CD45RA^+^CD62L^+^ cells compared to COR-treated mice (**Figure S7**). Moreover, T cells expressing CD62L in these cells were dominant in P4-treated mice compared to those in control and COR-treated mice. The expression level was higher in CD4 T cells compared to CD8 T cells. As for the other naïve marker, CD197 (47), the expression was not maintained after P4 treatment (**Figure 5C**). These results suggest that P4 maintains naïve and central memory T cell characteristics, while COR induces effector memory T cells, demonstrating the different functions of P4 and COR on T cell memory/effector differentiation.

If the T cells differentiate into effector memory cells, they may migrate into peripheral tissues. Therefore, we conducted IHC of human CD45^+^ cells in the lung. The results demonstrated that compared with P4-treated mice, COR-treated mice exhibited lung tissues with more infiltrated human leukocytes and with higher engraftment despite having the same donors (**Figure 5D**). These results suggest that the COR-treated T cells, which survive in this mouse system, tend to infiltrate peripheral tissues, while P4-treated T cells stay in the spleen.

### P4-treated T cells can maintain specific antibody production

**Figure 6:**
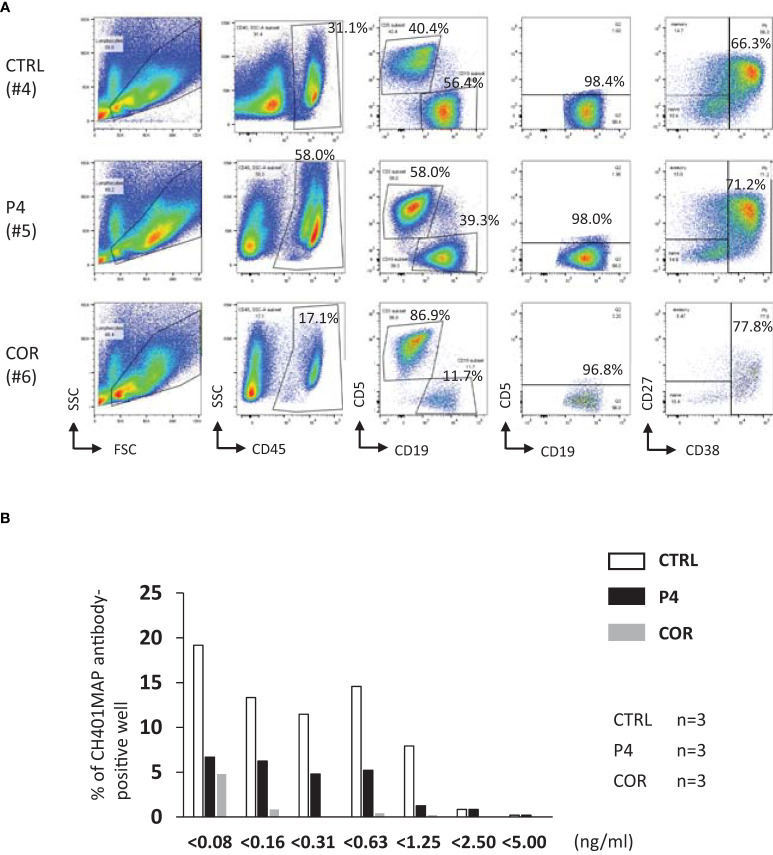
Effects of P4/COR pre-treatment on humoral immunity developed in humanized mice. The mouse spleen cells were examined for the phenotype of plasmablasts. The antibody-secreting B cell clones were detected by hybridoma formation. **(A)** Typical patterns of B cells engrafted in the mouse spleen. Spleen cells were gated for CD45^+^ cells, and the CD19^+^CD5- conventional B cells were selected. Finally, the expression of CD27 and CD38 were examined, and CD38^+^ cells were defined as plasmablasts. The ratio of CD38^+^ cells is shown in the panels. Mouse #4, #5, and #6 are referred to in **Table S3**. **(B)** The sum of hybridoma numbers obtained from three fusion experiments is shown. The horizontal axis showed the concentration of anti-CH401MAP IgGs detected from the supernatants of each well. Open bars; Control, Closed bars; P4 pre-treatment, Gray bars; COR pre-treated. Because the spleen cell number was insufficient, only three mouse spleens were used for the hybridoma preparation for COR pre-treated mice. So, as for the control and P4 pre-treatment, three mice were used. See also **Table S4**.

During pregnancy, mothers produce IgG antibodies and send them to the fetus. Based on the results that the T and B cells treated with P4 remain in the spleen, we hypothesized that the cells might produce functional IgG antibodies. Therefore, we immunized the humanized mice and examined the antigen-specific antibody production (**Table S3**). However, the titer in the antisera in humanized mice diverged individually, and no significant difference was detected. Meanwhile, most of the B cells detected in the humanized mice were plasmablasts, as we reported previously (42). The proportion of plasmablast was not different between P4 and COR-treated mice (**Figure 6A**). Therefore, we analyzed the production of antigen-specific B cell clones in the mice by preparing hybridomas and analyzing the whole antibodies and cross reactivity to the immunized antigen and third-party antigen. In COR-treated mice, the spleen cell number was low. Among the mice treated as shown in **Figure 5A**, only the mice with more than 1×10^7^ spleen cells were analyzed. We observed that whole IgG secreting clones were maintained in P4-treated mice, and with one exception, B cell clones of COR-treated mice also secreted IgGs (**Table S3**). On the contrary, P4-treated mice produced antigen-specific IgG secreting clones, a little less than control mice, but COR-treated mice did not produce antigen-specific B cell clones with high titer (**Figure 6B** and **Table S4**). These results suggest that transient treatment of lymphocytes with a high concentration of P4 maintains humanized mouse B cell function, while that of COR decreased the function significantly.

## Discussion

For a successful first and subsequent pregnancy, the immune environment of a mother needs to be profoundly changed to accept and maintain the semi-allogenic graft of the fetus. Furthermore, after delivery, alteration in immune conditions in the mother is essential to protect a mother’s body from the high risk of infection during the excretion of the allogeneic graft from the body. Reportedly, the important immune-regulatory steroid hormones P4 and COR increase during pregnancy (4, 7). P4, produced by trophoblast, shows a particularly significant alteration—trophoblasts secrete a large amount of P4 during the delivery, but the level of P4 drastically decreases after delivery. In this study, we analyzed the difference between the functions of P4 and COR in the context of mother/fetus immune modulation. We demonstrated that P4 suppressed the T cell function at the first stage of effector/memory development transiently and reversibly. By contrast, COR did not suppress the activation and differentiation of naïve T cells into activated/memory T cells. Nevertheless, COR suppressed both adaptive and innate immune cell-derived cytokines, which indicated that COR might eventually suppress the entire immune function. Moreover, our data revealed that P4 promoted T cell migration into lymphoid tissues and maintained T cell competency, suggesting the primacy of P4 relative to COR for the regulation of immune reaction against the allogeneic fetus.

P4 suppresses T cells activation in a concentration of placenta (48). We found that the suppression was reversible if the TCR signal was not induced in the presence of high dose P4. After the P4 pretreatment, not only CD4 T cells but also CD8 T cells accumulated in the spleen of humanized mice, and B cells produced antigen-specific IgG. Moreover, the lymphocytes could stay in the spleen of the mice. On the contrary, COR did not suppress activation of lymphocytes by TSST-1 and induced T cell proliferation and differentiation into effector/effector memory T cells. Instead, COR expressed similar level of exhaustion marker PD-1 on T cells compared to positive control. COR suppressed clone expansion of B cells secreting antigen-specific antibodies, eventually suppressing the specific antibody production. Furthermore, the secretion of inflammatory cytokines was significantly suppressed by COR compared to P4, which could be because COR is a strong immunosuppressor (49). Our study showed that P4 suppressed IL-10 production, as reported previously (50), while COR increased the production at 20 μM. The phenomenon suggests that P4 suppresses T cell differentiation into effector/memory cells at the first stage of naïve cells, and P4 does not induce a Treg-induced long-lived suppressive environment even if in the presence of COR. Moreover, in the presence of both steroids, the production of almost all inflammatory and T cell subset cytokines was suppressed. These results suggest that P4, largely secreted by the placenta, evades the effects of GC on T cells and supersedes the effect of COR. Therefore, it avoids irreversible suppression of adaptive immunity and maintains the secretion and transport of IgG antibodies to the fetus to protect it from pathogens.

Our study demonstrated that TCR-stimulated T cells died in the presence of a high concentration of P4. In a previous study, Th1 cells were reportedly killed by P4, but Th2 cells survived (51). Because T cells were not shifted to specific Th subset lineage in our system, the mechanism may differ from the previous report. However, the T cells stimulated in the presence of high dose P4, similar to the concentration of intervillous blood, are assumed to be a T cell group recognizing fetal antigens in the mother’s placenta. Therefore, the death of the T cells before proliferation and differentiation into Th subset lineages confers a great advantage for protecting the fetus. Nevertheless, it is very important for the T cells not stimulated in the high-P4 concentration to survive and function normally in the low P4 environment. Our results indicate that the extensive P4 production in the limited area of the placenta constructs an environment that is beneficial for pregnant immunity.

Several studies have compared the progesterone receptor (PR) and GC receptor (GCR) and demonstrated that the receptors are not specific, and that P4 can suppress GC by binding to GCR (10, 52). Therefore, P4 and COR have been thought to function similarly in the immune system. However, our results showed a clear difference between the two steroids. Although the effects of P4 on innate immunity were not larger than COR (based on the cytokine production), its effects on T cells stimulated with TCR were prominent. This result is consistent with that of a previous study (24). Furthermore, our results showed that the only P4 treatment group suppressed the expression of activation markers and cell proliferation. The group exhibited neither the development of TSCM cells, the earliest memory cells of T cells (53, 54), nor the secretion of cytokines. Because the suppression is caused in the earliest phase in the naïve T cell differentiation into effector/memory cells, the effect of P4 might be non-genetic. It is not likely that the steroid receptors are the main signal transducer for the non-genetic function. Previously, P4 was reported to bind to the Kv1.3, a potassium channel, to suppress T cell activation (24). Kv1.3 inhibition decreases Ca^2+^ influx, which induces apoptosis by mitochondria function defect (55). Combined with the above discussion that the TCR-stimulated T cells die in the presence of high dose P4, P4 is suggested to inhibit Kv1.3 on the T cells. As the suppressive affinity of P4 is higher than COR, in line with a previous report (24), it is rational that P4 suppression is dominant compared to COR proliferation. In the mouse immune system, the Kv1.3 channel is reported not to be important in T cell activation (56), which suggests the benefit of using a humanized mouse system for the analysis of P4 non-genetic function. Moreover, the difference between males and females was not observed in the *in vitro* assay. The finding indicates that the difference is not caused by the expression of hormonal receptors. However, further analyses of the molecular mechanisms are necessary to clarify the difference in detail by using an *in vitro* culture system and the humanized pregnant immune system, because the re-construction of the immune environment in the placenta is difficult in this simple culture system.

COR is reported to induce B cell maturation into antibody-secreting cells (57). Although the number of engrafted human CD45^+^ cells was lower in COR-treated mice than in control and P4-treated mice, some of the COR-treated mice possessed a high level of specific antibodies in the sera (**Table S3**). In addition, antigen-non-specific IgG-secreting clones were detected. Therefore, non-specific antibody-secreting B cells might become plasmablasts by the 6 h pre-treatment of COR. This might be because COR induces B lymphocyte-induced maturation protein-1 (BLIMP-1) expression in the B cells, developing plasma cells irrespective of the stimulation by antigens. Contrary to this finding, very few antigen-specific clones per spleen cells were detected in COR-treated mice. The reason might be the low proportion of B cells in the spleen CD45^+^ cells. The defects of antigen-specific plasmablast formation might be caused by the defect of functional B cell engraftment by COR pre-treatment. Irreversible and systemic humoral immune suppression has no advantage for pregnant immunity. Accordingly, neutralizing the effects of COR with a large amount of P4 may be necessary to sustain pregnancy.

Our study showed that T cells migrate to peripheral tissues in the presence of COR, which contradicts previous studies, which have shown that physiological concentration of COR enhances IL-7 receptor (CD127) expression on T cells to induce homing to lymphoid tissues by regulating chemokine receptor expression (31, 57). We also observed an increase in CD127 expression together with CD197 expression in the COR-mouse T cells. However, as human IL-7 is not supplied to T cells in our humanized mouse system, they may not acquire adequate signals from the IL-7 receptors. However, after 4 weeks, COR-pre-treated T cells engrafted in the mice did not show any difference in CD197 expression compared to control or P4 pre-treated mice (data not shown), suggesting that 6 h treatment of high dose COR might not maintain the expression of the homing receptor.

**Figure 7:**
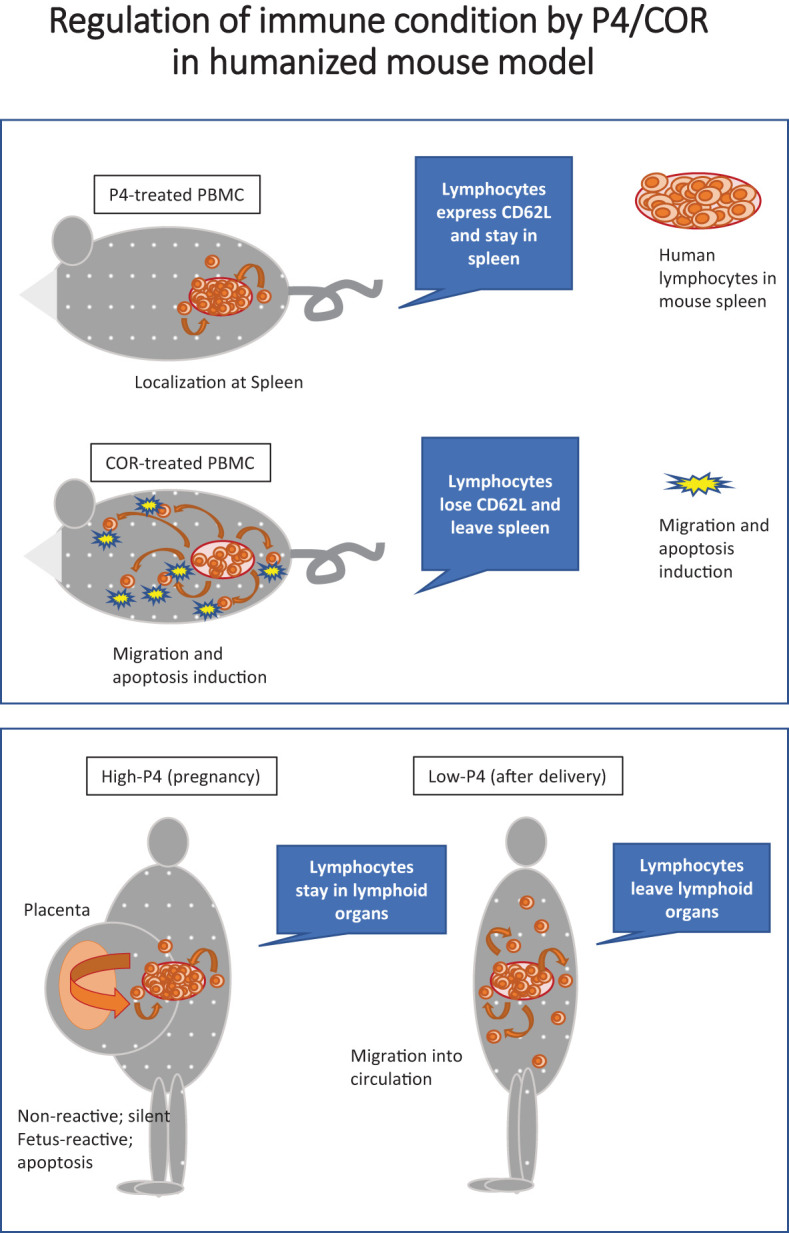
Model for pregnant immune system affected by P4/COR *in vivo*. Left panel; Regulation of immune condition by P4/COR in the humanized mouse model (NOG-hIL-4-Tg). P4 treatment maintains CD62L expression on T cells and contributes to T cell accumulation in the spleen. In contrast, COR does not maintain the CD62L expression, and the T cell activation and migration in the periphery cannot be suppressed. Right panel; Regulation of immune condition by P4/COR in human pregnancy. A high P4 environment in the placenta maintains non-reactive T cells but kills fetus-reactive T cells. The naïve T cells are stored in the peripheral lymphoid tissues such as the spleen and lymph node. However, after the delivery, P4 concentration drastically decreases, and the stored naïve T cells migrate into the periphery, protecting the mother from infectious disease.

We also found that a significant portion of P4-treated T cells maintained the expression of CD62L in the humanized mouse spleens for four weeks, which could be a genetic effect of P4 and not affected by IL-7 existence. This is comparable with the fact that CD62L expression needs FOXO1 expression (58) — FOXO1 is induced by P4, wherein the FOXO1-binding site is found in the CD62L promoter, which may explain the supportive role of P4 in the positive regulation of CD62L. However, it is surprising that only 6 h treatment of PBMCs maintains the CD62L expression. The treatment sustained a significant number of central memory T cells in the spleen (**Figure 7**, upper panel). It might avoid the inflammation of the peripheral tissues and contribute to the production of antigen-specific antibodies, which would be sent to the fetus. Therefore, P4 might not be only a non-genetic Kv1.3 inhibitor to preserve naïve T cells in the lymph nodes but also an enhancer of central memory T cell differentiation and IgG production.

The preserved expression of CD62L might partly explain the transient increase of naïve T cells in the maternal circulation just after the delivery because P4 preserves the T cells in the lymphatic tissues along with the inhibition of activation (**Figure 7**, lower panel). However, it is controversial because the CD62L expression on T cells was prolonged for four weeks after the 6 h-treatment of high dose P4 in humanized mice, while CD197 expression was decreased in the presence of P4. Although CD197 is also a migration marker to lymphoid tissues (47), the role of CD197 is reported to control T cell motility in the lymph node (59), suggesting that the naïve T cells are maintained in the absence of CD197 in the lymph node. Meanwhile, the CD197 signal inhibits T cell proliferation (60). Therefore, the cells might proliferate without CD197, which might be beneficial to keeping naïve or very early memory cell numbers in the pregnant woman. Other signals lacking in humanized mice may be needed to mobilize naïve T cells stored in the lymphoid tissues after the delivery.

Another migration-related molecule expressed on T cells is sphingosine 1-phosphate receptor-1 (S1P-1) that promotes both egress of T cells (61), and T cell migration between non-lymphoid tissues and lymphatics (62). While CD197 counteracts with S1P receptor-1 (S1PR1), humanized mice tissues express only mouse CCL19 or CCL21, which might not react with human CD197. Therefore, human T cells that express S1PR1 might egress lymphatic organs but not be suppressed by CD197 signals because the ligands expressed on the tissues are mouse molecules. Recently, Xiong et al. reported that blocking the signaling pathway of S1P-1 induced immune tolerance and child-loss was prevented (63). In the absence of a CD197 signal, P4 might support the function of immunotolerance by a different homing mechanism from S1P-1 and/or CD197 signals by using CD62L.

Collectively, this study clarified that high P4 environment caused transient and reversible suppression of T cell activation while COR did not. P4 maintained CD62L expression on T cells and promoted the T cell migration in the spleen and the preservation of humoral immunity, while COR did not. These functional differences might modulate the immunity of the pregnant mother to protect the semi-allogenic fetus and to maintain anti-pathogen and anti-cancer immunity.

Although the phenomenon explains in part the transient increase of naïve T cells in the PBs of pregnant women just after delivery, our humanized mouse system did not completely mimic the human pregnant environment (1). The naïve phenotype of T cells was not maintained. (2) The mouse system largely maintains T cells and B cells, and very few innate immune cells are engrafted. (3) The expression of homing receptors is insufficient. (4) Lymph nodes are not formed in the periphery. Therefore, the contribution of naïve T cells and other APCs should be evaluated using other systems. Moreover, T cell homing should be evaluated by developing a new humanized mouse model expressing homing-related factors such as CD197 ligands of human origin.

Further analysis may clarify the molecular mechanism of how P4 controls T cell arrest in a naïve state with CD197 downregulation. Especially, it is important to clarify which kind of steroid receptors are engaged in this function. The benefit of naïve T cell reservation in the lymph node will also be clarified using an improved humanized mouse system in the future.

## Data availability statement

The raw data supporting the conclusions of this article will be made available by the authors, without undue reservation.

## Ethics statement

The studies involving human participants were reviewed and approved by Tokai University Human Research Committee. The patients/participants provided their written informed consent to participate in this study. The animal study was reviewed and approved by Institutional Animal Care and Use Committee at Tokai University.

## Author contributions

HK, TOSS, and YK contributed to the conception and design of the study. RI, TOSS, and YK contributed to the development of the methodology. HK, SO, YO, TOMS, SY, NK, YG, and AY contributed to the acquisition and analysis of data. HK, TOSS, and YK contributed to the writing, review, and/or revision of the manuscript. HK, TAKS, YG, RI, and BT provided administrative, technical, or material support. S-II, HI, TAKS, and YK supervised the study. All authors contributed to the article and approved the submitted version.

## Funding

This work was supported by MEXT KAKENHI (Grant Number 22220007; Mamoru Ito, 17H03571; YK), AMED (Grant Number JP18lm0203004, JP19lm0203004, JP20lm0203004, JP21lm0203004), Tokai University School of Medicine Project Research to YK (2013–2015), and Tokai University Grant-in-Aid to YK (2015–2017).

## Acknowledgments

The authors thank the members of the Teaching and Research Support Center in Tokai University School of Medicine for their technical skills. We also thank Yumiko Nakagawa for her excellent animal care skills.

## Conflict of interest

The authors declare that the research was conducted in the absence of any commercial or financial relationships that could be construed as a potential conflict of interest.

## Publisher’s note

All claims expressed in this article are solely those of the authors and do not necessarily represent those of their affiliated organizations, or those of the publisher, the editors and the reviewers. Any product that may be evaluated in this article, or claim that may be made by its manufacturer, is not guaranteed or endorsed by the publisher.

## References

[1] Di Toro F Gjoka M Di Lorenzo G De Santo D De Seta F Maso G. Impact of covid-19 on maternal and neonatal outcomes: A systematic review and meta-analysis. Clin Microbiol Infect (2021) 27(1):36–46. doi: 10.1016/j.cmi.2020.10.007 33148440 PMC7605748

[2] Wastnedge EAN Reynolds RM van Boeckel SR Stock SJ Denison FC Maybin JA. Pregnancy and covid-19. Physiol Rev (2021) 101(1):303–18. doi: 10.1152/physrev.00024.2020 PMC7686875 32969772

[3] Daniilidis A Giannoulis C Sardeli C Dinas K Nasioutziki M Tantanasis T. Pregnancy-associated breast cancer–a review analysis. Eur J Gynaecol Oncol (2010) 31(5):485–90. 21061786

[4] Morel Y Roucher F Plotton I Goursaud C Tardy V Mallet D. Evolution of steroids during pregnancy: Maternal, placental and fetal synthesis. Ann Endcriol (2016) 77:82–9. doi: 10.1016/j.ando.2016.04.023 27155772

[5] Mastorakos G Ilias I. Maternal and fetal hypothalamic-Pituitary-Adrenal axes during pregnancy and postpartum. Ann N Y Acad Sci (2003) 997:136–49. doi: 10.1196/annals.1290.016 14644820

[6] Tuckey RC. Progesterone synthesis by the human placenta. Placenta (2005) 26(4):273–81. doi: 10.1016/j.placenta.2004.06.012 15823613

[7] Chatuphonprasert W Jarukamjorn K Ellinger I. Physiology and pathophysiology of steroid biosynthesis, transport and metabolism in the human placenta. Front Pharmacol (2018) 9:1027. doi: 10.3389/fphar.2018.01027 30258364 PMC6144938

[8] Stites D Bugbee S Siiteri P. Differential actions of progesterone and cortisol on lymphocyte and monocyte interaction during lymphocyte activation–relevance to immunosuppression in pregnancy. J Reprod Immunol (1983) 5(4):215–28. doi: 10.1016/0165-0378(83)90237-1 6604810

[9] Stites D Siiteri P. Steroids as immunosuppressants in pregnancy. Immunol Rev (1983) 75:117–38. doi: 10.1111/j.1600-065X.1983.tb01093.x 6226589

[10] Hierweger AM Engler JB Friese MA Reichardt HM Lydon J DeMayo F. Progesterone modulates the T-cell response *Via* glucocorticoid receptor-dependent pathways. Am J Reprod Immunol (2019) 81(2):e13084. doi: 10.1111/aji.13084 30604567 PMC7457140

[11] Mestas J Hughes C. Of mice and not men: Differences between mouse and human immunology. J Immunol (2004) 172(5):2731–8. doi: 10.4049/jimmunol.172.5.2731 14978070

[12] Ito R Takahashi T Katano I Ito M. Current advances in humanized mouse models. Cell Mol Immunol (2012) 9(3):208–14. doi: 10.1038/cmi.2012.2 PMC4012844 22327211

[13] Walsh NC Kenney LL Jangalwe S Aryee KE Greiner DL Brehm MA. Humanized mouse models of clinical disease. Annu Rev Pathol (2017) 12:187–215. doi: 10.1146/annurev-pathol-052016-100332 27959627 PMC5280554

[14] Arck P Hansen PJ Mulac Jericevic B Piccinni MP Szekeres-Bartho J. Progesterone during pregnancy: Endocrine-immune cross talk in mammalian species and the role of stress. Am J Reprod Immunol (2007) 58(3):268–79. doi: 10.1111/j.1600-0897.2007.00512.x 17681043

[15] Kashiwagi H Ishimoto H Izumi S Seki T Kinami R Otomo A. Human pzp and common marmoset A2ml1 as pregnancy related proteins. Sci Rep (2020) 10(1):5088. doi: 10.1038/s41598-020-61714-8 32198464 PMC7083932

[16] Hodges JK Henderson C Hearn JP. Relationship between ovarian and placental steroid production during early pregnancy in the marmoset monkey (Callithrix jacchus). J Reprod Fertil (1983) 69(2):613–21. doi: 10.1530/jrf.0.0690613 6415278

[17] Carter AM. Unique aspects of human placentation. Int J Mol Sci (2021) 22(15):8099. doi: 10.3390/ijms22158099 34360862 PMC8347521

[18] Carter AM Enders AC Pijnenborg R. The role of invasive trophoblast in implantation and placentation of primates. Philos Trans R Soc Lond B Biol Sci (2015) 370(1663):20140070. doi: 10.1098/rstb.2014.0070 25602074 PMC4305171

[19] Runnebaum B Runnebaum H Stober I Zander J. Progesterone 20 alpha-dihydroprogesterone and 20 beta-dihydroprogesterone levels in different compartments from the human foeto-placental unit. Acta Endocrinol (1975) 80:558–68. doi: 10.1530/acta.0.0800558 1242570

[20] Runnebaum B Stober I Zander J. Progesterone, 20 alpha-dihydroprogesterone and 20 beta-dihydroprogesterone in mother and child at birth. Acta Endocrinol (1975) 80:569–76. doi: 10.1530/acta.0.0800569 1242571

[21] Szekeres-Bartho J Autran B Debre P Andreu G Denver L Chaouat G. Immunoregulatory effects of a suppressor factor from healthy pregnant women’s lymphocytes after progesterone induction. Cell Immunol (1989) 122(2):281–94. doi: 10.1016/0008-8749(89)90077-4 2527616

[22] Papapavlou G Hellberg S Raffetseder J Brynhildsen J Gustafsson M Jenmalm MC. Differential effects of estradiol and progesterone on human T cell activation *in vitro* . Eur J Immunol (2021) 51(10):2430–40. doi: 10.1002/eji.202049144 34223649

[23] Hunt JS Petroff MG McIntire RH Ober C. Hla-G and immune tolerance in pregnancy. FASEB J (2005) 19(7):681–93. doi: 10.1096/fj.04-2078rev 15857883

[24] Ehring G Kerschbaum H Eder C Neben A Fanger C Khoury R. A nongenomic mechanism for progesterone-mediated immunosuppression: Inhibition of k+ channels, Ca2+ signaling, and gene expression in T lymphocytes. J Exp Med (1998) 188:1593–602. doi: 10.1084/jem.188.9.1593 PMC2212508 9802971

[25] Hellberg S Raffetseder J Rundquist O Magnusson R Papapavlou G Jenmalm MC. Progesterone dampens immune responses in *in vitro* activated Cd4(+) T cells and affects genes associated with autoimmune diseases that improve during pregnancy. Front Immunol (2021) 12:672168. doi: 10.3389/fimmu.2021.672168 34054852 PMC8149943

[26] Pfefferle D Brauch K Heistermann M Hodges JK Fischer J. Female Barbary macaque (Macaca sylvanus) copulation calls do not reveal the fertile phase but influence mating outcome. Proc Biol Sci (2008) 275(1634):571–8. doi: 10.1098/rspb.2007.1499 PMC2596815 18089536

[27] Ashwell JD Lu FW Vacchio MS. Glucocorticoids in T cell development and function*. Annu Rev Immunol (2000) 18:309–45. doi: 10.1146/annurev.immunol.18.1.309 10837061

[28] Taves M Ashwell J. Glucocorticoids in T cell development, differentiation and function. Nat Rev | Immunol (2021) 21:233–43. doi: 10.1038/s41577-020-00464-0 33149283

[29] Magiakou MA Mastorakos G Rabin D Margioris AN Dubbert B Calogero AE. The maternal hypothalamic-Pituitary-Adrenal axis in the third trimester of human pregnancy. Clin Endocrinol (Oxf) (1996) 44(4):419–28. doi: 10.1046/j.1365-2265.1996.683505.x 8706308

[30] Mastorakos G Ilias I. Maternal hypothalamic-Pituitary-Adrenal axis in pregnancy and the postpartum period. Postpartum-Related Disord Ann N Y Acad Sci (2000) 900:95–106. doi: 10.1111/j.1749-6632.2000.tb06220.x 10818396

[31] Shimba A Cui G Tani-Ichi S Ogawa M Abe S Okazaki F. Glucocorticoids drive diurnal oscillations in T cell distribution and responses by inducing interleukin-7 receptor and Cxcr4. Immunity (2018) 48(2):286–98.e6. doi: 10.1016/j.immuni.2018.01.004 29396162

[32] Yang K. Placental 11 beta-hydroxysteroid dehydrogenase: Barrier to maternal glucocorticoids. Rev Reprod (1997) 2(3):129–32. doi: 10.1530/ror.0.0020129 9414475

[33] Valsamakis G Chrousos G Mastorakos G. Stress, female reproduction and pregnancy. Psychoneuroendocrinology (2019) 100:48–57. doi: 10.1016/j.psyneuen.2018.09.031 30291988

[34] Sahr A Former S Hildebrand D Heeg K. T-Cell activation or tolerization: The yin and yang of bacterial superantigens. Front Microbiol (2015) 6:1153. doi: 10.3389/fmicb.2015.01153 26539181 PMC4611159

[35] Taylor A Llewelyn M. Superantigen-induced proliferation of human Cd4+Cd25- T cells is followed by a switch to a functional regulatory phenotype. J Immunol (2010) 185(11):6591–8. doi: 10.4049/jimmunol.1002416 21048104

[36] Alouf JE Muller-Alouf H. Staphylococcal and streptococcal superantigens: Molecular, biological and clinical aspects. Int J Med Microbiol (2003) 292(7-8):429–40. doi: 10.1078/1438-4221-00232 12635926

[37] Verhaar AP Wildenberg ME Duijvestein M Vos AC Peppelenbosch MP Lowenberg M. Superantigen-induced steroid resistance depends on activation of phospholipase Cbeta2. J Immunol (2013) 190(12):6589–95. doi: 10.4049/jimmunol.1202898 23690479

[38] Shultz L Brehm M Garcia-Martinez J Greiner D. Humanized mice for immune system investigation: Progress, promise and challenges. Nat Rev Immunol (2012) 12(11):786–98. doi: 10.1038/nri3311 PMC3749872 23059428

[39] Ito M Hiramatsu H Kobayashi K Suzue K Kawahata M Hioki K. Nod/Scid/Gamma(C)(Null) mouse: An excellent recipient mouse model for engagement of human cells. Blood (2002) 100:3175–82. doi: 10.1182/blood-2001-12-0207 12384415

[40] Kametani Y Ohno Y Ohshima S Tsuda B Yasuda A Seki T. Humanized mice as an effective evaluation system for peptide vaccines and immune checkpoint inhibitors. Int J Mol Sci (2019) 20(24):6337. doi: 10.3390/ijms20246337 PMC6940818 31888191

[41] Kametani Y Katano I Miyamoto A Kikuchi Y Ito R Muguruma Y. Nog-Hil-4-Tg, a new humanized mouse model for producing tumor antigen-specific igg antibody by peptide vaccination. PloS One (2017) 12(6):e0179239. doi: 10.1371/journal.pone.0179239 28617827 PMC5472286

[42] Ohno Y Ohshima S Miyamoto A Kametani F Ito R Tsuda B. Her2-Antigen-Specific humoral immune response in breast cancer lymphocytes transplanted in hu-pbl hil-4 nog mice. Sci Rep (2021) 11(1):12798. doi: 10.1038/s41598-021-92311-y 34140620 PMC8211648

[43] Miyako H Kametani Y Katano I Ito R Tsuda B Furukawa A. Antitumor effect of new Her2 peptide vaccination based on b cell epitope. Anticancer Res (2011) 31(10):361–3368. 21965747

[44] Grailer JJ Kodera M Steeber DA. L-selectin: Role in regulating homeostasis and cutaneous inflammation. J Dermatol Sci (2009) 56(3):141–7. doi: 10.1016/j.jdermsci.2009.10.001 PMC2787637 19889515

[45] Konno O Hirano T Katsuyama K Oka K Matsuno N Nagao T. Bacterial superantigen tsst-1 attenuates suppressive efficacy of glucocorticoids and calcineurin inhibitors against blastogenesis of peripheral blood mononuclear cells from patients with chronic renal failure on hemodialysis treatment. Transpl Immunol (2007) 17(3):187–92. doi: 10.1016/j.trim.2006.10.003 17331845

[46] Kondo T Ando M Nagai N Tomisato W Srirat T Liu B. The notch-Foxm1 axis plays a key role in mitochondrial biogenesis in the induction of human stem cell memory-like car-T cells. Cancer Res (2020) 80(3):471–83. doi: 10.1158/0008-5472.CAN-19-1196 31767627

[47] Sallusto F Lenig D Forster R Lipp M Lanzavecchia A. Two subsets of memory T lymphocytes with distinct homing potentials and effector functions. Nature (1999) 401(6754):708–12. doi: 10.1038/44385 10537110

[48] Shah NM Lai PF Imami N Johnson MR. Progesterone-related immune modulation of pregnancy and labor. Front Endocrinol (Lausanne) (2019) 10:198. doi: 10.3389/fendo.2019.00198 30984115 PMC6449726

[49] Ehrchen JM Roth J Barczyk-Kahlert K. More than suppression: Glucocorticoid action on monocytes and macrophages. Front Immunol (2019) 10:2028. doi: 10.3389/fimmu.2019.02028 31507614 PMC6718555

[50] Bommer I Muzzio DO Zygmunt M Jensen F. Progesterone and estradiol exert an inhibitory effect on the production of anti-inflammatory cytokine il-10 by activated mz b cells. J Reprod Immunol (2016) 116:113–6. doi: 10.1016/j.jri.2016.05.008 27317920

[51] Matsuzaki M Takemasa Tsuji T Imazeki I Ikeda H Nishimura T. Immunosteroid as a regulator for Th1/Th2 balance: Its possible role in autoimmune diseases. Autoimmunity (2005) 38:369–75. doi: 10.1080/08916930500124122 16227152

[52] Kontula K Paavonen T Luukkainen T Andersson LC. Binding of progestins to the glucocorticoid receptor. Correlation to their glucocorticoid-like effects on *in vitro* functions of human mononuclear leukocytes. Biochem Pharmacol (1983) 32(9):1511–8. doi: 10.1016/0006-2952(83)90474-4 6222739

[53] Fearon DT Manders P Wagner SD. Arrested differentiation, the self-renewing memory lymphocyte, and vaccination. Science (2001) 293(5528):248–50. doi: 10.1126/science.1062589 11452114

[54] Gattinoni L Lugli E Ji Y Pos Z Paulos CM Quigley MF. A human memory T cell subset with stem cell-like properties. Nat Med (2011) 17(10):1290–7. doi: 10.1038/nm.2446 PMC3192229 21926977

[55] Checchetto V Prosdocimi E Leanza L. Mitochondrial Kv1.3: A new target in cancer biology? Cell Physiol Biochem (2019) 53(S1):52–62. doi: 10.33594/000000195 31854954

[56] Ishida Y Chused TM. Lack of voltage sensitive potassium channels and generation of membrane potential by sodium potassium atpase in murine T lymphocytes. J Immunol (1993) 151(2):610–20. 8393035

[57] Franco L Gadkari M Howe K Sun J Kardava L Kumar P. Immune regulation by glucocorticoids can be linked to cell type-dependent transcriptional responses. J Exp Med (2019) 216(2):384–406. doi: 10.1084/jem.20180595 30674564 PMC6363437

[58] Lou Y Lu X Dang X. Foxo1 up-regulates human l-selectin expression through binding to a consensus Foxo1 motif. Gene Regul Syst Biol (2012) 6:139–49. doi: 10.4137/GRSB.S10343 PMC3486891 23133314

[59] Asperti-Boursin F Real E Bismuth G Trautmann A Donnadieu E. Ccr7 ligands control basal T cell motility within lymph node slices in a phosphoinositide 3-Kinase-Independent manner. J Exp Med (2007) 204(5):1167–79. doi: 10.1084/jem.20062079 PMC2118589 17485513

[60] Ziegler E Oberbarnscheidt M Bulfone-Paus S Förster R Kunzendorf U Krautwald S. Ccr7 signaling inhibits T cell proliferation. J Immunol (2007) 10):6485–93. doi: 10.4049/jimmunol.179.10.6485 17982037

[61] Pham T Okada T Matloubian M Lo C Cyster J. S1p1 receptor signaling overrides retention mediated by G alpha I-coupled receptors to promote T cell egress. Immunity (2008) 28(1):122–33. doi: 10.1016/j.immuni.2007.11.017 PMC2691390 18164221

[62] Baeyens A Fang V Chen C Schwab SR. Exit strategies: S1p signaling and T cell migration. Trends Immunol (2015) 36(12):778–87. doi: 10.1016/j.it.2015.10.005 PMC4832571 26596799

[63] Xiong M Xu L Li L Liu Y Zhou F Wang J. The experimental research of pregnancy immune tolerance induced by Fty720 *Via* blocking S1p signal transduction pathway. J Cell Biochem (2019) 120(4):5897–905. doi: 10.1002/jcb.27876 30362168

