# Supplementary material for: High-progesterone environment preserves T cell competency by evading glucocorticoid effects on immune regulation
Source: Front Immunol. 2022 Sep 20;13:1000728. doi: 10.3389/fimmu.2022.1000728 (PMC9530059; doi:10.3389/fimmu.2022.1000728)
Supplement: Supplementary file 1 [file DataSheet_1.pdf]

Table S1

Sample name	Delivery type	Delivery date	Date of blood sampling		
			Pre-delivery	Post-delivery day	1Mo after delivery
P1	Scheduled-C/S	37w6d	-1	4	35
P2	Transvaginal Delivery	40w2d	-1	4	35
P3	Transvaginal Delivery	40w5d	0	4	34
P4	Transvaginal Delivery	40w2d	0	4	36
P5	Transvaginal Delivery	39w3d	-1	4	27
P6	Emergency-C/S	40w1d	-1	4	29
P7	Transvaginal Delivery	40w5d	-1	4	36
P8	Transvaginal Delivery	40w1d	-3	4	37
P9	Transvaginal Delivery	40w3d	0	4	28
P10	Emergency-C/S	36w5d	0	4	32
P11	Emergency-C/S	40w0d	-2	3	39
P12	Emergency-C/S	39w3d	-1	4	28
P13	Transvaginal Delivery	25w1d	0	3	27
P14	Scheduled-C/S	38w0d	-1	3	35
P15	Emergency-C/S	31w2d	0	3	32

Table S1 Clinical data of pregnant women. The delivery type, date and the date of blood sampling was shown. The date of delivery is shown as 0.

Table S2

Antibody	Clone	Company	Cat.No.
FITC anti-human CD3	UCHT1	BioLegend	300406
APC anti-human CD3	SK7	BD Bioscience	340440
APC/Cy7 anti-human CD3	SK7	BioLegend	344818
APC anti-human CD4	RPA-T4	BioLegend	300514
BrilliantViolet510TM anti-human CD4	OKT4	BioLegend	317444
Alexa Fluor®700 anti-human CD4	OKT4	eBioscience	317426
PE/Cy7 anti-human CD5	UCHT2	BioLegend	300622
Alexa Fluor®700 anti-human CD8	HIT8a	BioLegend	300920
BV395 anti-human CD8	RPA-T8	Biosciences	563795
APC/Cy7 anti-human CD19	HIB19	BioLegend	302218
PE anti-human CD25	BC96	BioLegend	302606
PE/Cy7 anti-human CD25	BC96	BioLegend	302612
PE anti-human CD27	M-T271	BD Bioscience	555441
Alexa Fluor®700 anti-human CD38	HIT2	BioLegend	303524
Pacific BlueTM anti-human CD45	HI30	BioLegend	304022
BUV737 anti-human CD45	HI30	Biosciences	748719
BrilliantViolet650TM anti-human CD45RA	HI100	BioLegend	304136
APC/Cy7 anti-human CD45RO	UCHL1	BioLegend	304228
PE anti-human CD62L	DREG-56	BioLegend	304806
BV421 anti-human CD95	DX2	BioLegend	305624
PE/Cy7 anti-human CD127	A01905	BioLegend	351320
BrilliantViolet510TM anti-human CD127 (IL-7Rα)	A019D5	BioLegend	351332
PerCP/Cy5.5 anti-human CD183 (CXCR3)	G025H7	BioLegend	353714
APC anti-human CD197 (CCR7)	G043H7	BioLegend	353214
PerCP/Cy5.5 anti-human CD279 (PD-1)	EH12.2H7	BioLegend	329914

Table S2 List of antibodies used for the analysis. The clone numbers , company and the catalog numbers are shown.

Table S3

CTRL

mouse #	serum anti-CH401MAP conc (ng/ml)	h-IL-4 conc. (pg/mL)	mean colony #/well	anti-CH401MAP conc (ng/ml) in mice	hIgG+well #	anti-CH401MAP +well #	hIgG+well (%)	anti-CH401MAP +well (%)	SPL cell # ($x10^7$)	CD45(%)	CD19/CD45(%)	CD27+CD38+/ CD19 (%)	CD19/CD45+ (%)	CD19+ CD27+ C38+ /CD45(%)
#1	10.1	209.0	3.2	27.54	*96/96	74/96	100.0%	67.7%	8.3	39.4	42.1	38.9	16.6	6.46
#4	36.1	300.6	19.3	10.43	*96/96	91/96	100.0%	94.8%	6.3	39.1	63.3	69.4	24.8	17.18
#7	8.3	267.6	10.3	8.13	*96/96	96/96	100.0%	100.0%	6.7	77.6	23.2	64.8	18.0	11.67
#10	9.0	201.1	16.1	19.68	*96/96	63/96	100.0%	65.6%	5.4	36.1	46.5	67.0	16.8	11.25

P4

mouse #	serum anti-CH401MAP conc (ng/ml)	h-IL-4 conc. (pg/mL)	mean colony #/well	anti-CH401MAP conc (ng/ml) in mice	hIgG+well #	anti-CH401MAP +well #	hIgG+well (%)	anti-CH401MAP +well (%)	SPL cell # ($x10^7$)	CD45(%)	CD19/CD45(%)	CD27+CD38+/ CD19 (%)	CD19/CD45+ (%)	CD19+ CD27+ C38+ /CD45+(%)
#2	114.8	138.4	6.4	5.9	*96/96	3/96	100.0%	3.1%	4.4	34.9	40.9	31.7	14.3	4.52
#5	36.1	210.6	17.5	71.57	*96/96	64/96	100.0%	66.7%	16.1	61.5	44	71	27.1	19.21
#8	8.3	243.3	18.3	37.25	*96/96	46/96	100.0%	47.9%	16.6	51.5	31.1	71.3	16.0	11.42
#11	9.0	192.8	6.5	2.95	*96/96	8/96	100.0%	8.3%	1.7	16.2	39.8	71.9	6.4	4.64

COR

mouse #	serum anti-CH401MAP conc (ng/ml)	h-IL-4 conc. (pg/mL)	mean colony #/well	anti-CH401MAP conc (ng/ml) in mice	hIgG+well #	anti-CH401MAP +well #	hIgG+well (%)	anti-CH401MAP +well (%)	SPL cell # ($x10^7$)	CD45(%)	CD19/CD45(%)	CD27+CD38+/ CD19 (%)	CD19/CD45+ (%)	CD19+ CD27+ C38+ /CD45+(%)
#3	10.1	240.2	3.1	0.40	*94/96	23/96	97.9%	24.0%	1.7	3.0	9.7	6.9	0.3	0.02
#6	36.1	109.9	3.8	219.86	*96/96	1/96	100.0%	1.0%	5.6	25.1	21.2	85.3	5.3	4.54
#9	10.6	234.1	5.7	0.00	*3/96	0/96	3.1%	0.0%	7.3	0.2	15.1	0	0.0	0.00
#12	8.3	255.1	11.0	4.39	*96/96	6/96	100.0%	6.3%	5.4	69.4	26	65.5	18.0	11.82

Table S3 Hybridoma yield of B cell clone secreting antigen-specific antibody on humanized mouse spleen cells. Upper table; Control mice, middle table; P4-treated mice, Lower table; COR treated mice. #; Individual mouse number. Serum anti-CH401MAP conc.; The specific antibody amount and total human IgG amount involved in the mouse plasma collected 28 days after the transplantation of PBMC. h-IL-4 conc.; The human IL-4 level in the plasma of each mouse collected at 4th week after birth. Mean colony number/ well; All the clones detected in the wells were counted and the average was calculated. hIgG+ well #; The wells in which human total IgG was detected were counted. Anti-CH401MAP+ well #; The wells in which anti-CH401MAP IgG was detected, were counted. hIgG+ well %; The number of total IgG+ well /total wells. Anti-CH401MAP+ well %; The number of Anti-CH401MAP+ well /total wells. Total number of CH401MAP-specific IgG- positive well, %; The percentage of CH401MAP specific IgG-positive well per total well number. All the data are sum of three independent experiments.
* Supernatants were diluted 1:11 and assayed by ELISA.

Table S4

Concentration (ng/ml)	CTRL (n=3)		P4 (n=3)		COR (n=3)	
	Number	%	Number	%	Number	%
<0.08	92	19.2	32	6.7	23	4.8
<0.16	64	13.3	30	6.3	4	0.8
<0.31	55	11.5	23	4.8	0	0.0
<0.63	70	14.6	25	5.2	2	0.4
<1.25	38	7.9	6	1.3	1	0.2
<2.5	4	0.8	4	0.8	0	0.0
<5	1	0.2	1	0.2	0	0.0
**SUM**	**324**	**67.5**	**121**	**25.2**	**30**	**7.8**

Table S4 Specific antibody-secreting clone yield on steroid-treated human B cells engrafted in NOG-hIL-4-Tg mouse. Number; Total number of CH401MAP-specific IgG- positive well, %; The percentage of CH401MAP specific IgG-positive well per total well number (The data is a sum of three independent experiments. The number of wells with and without any specific antibodies are involved.).

Figure S1

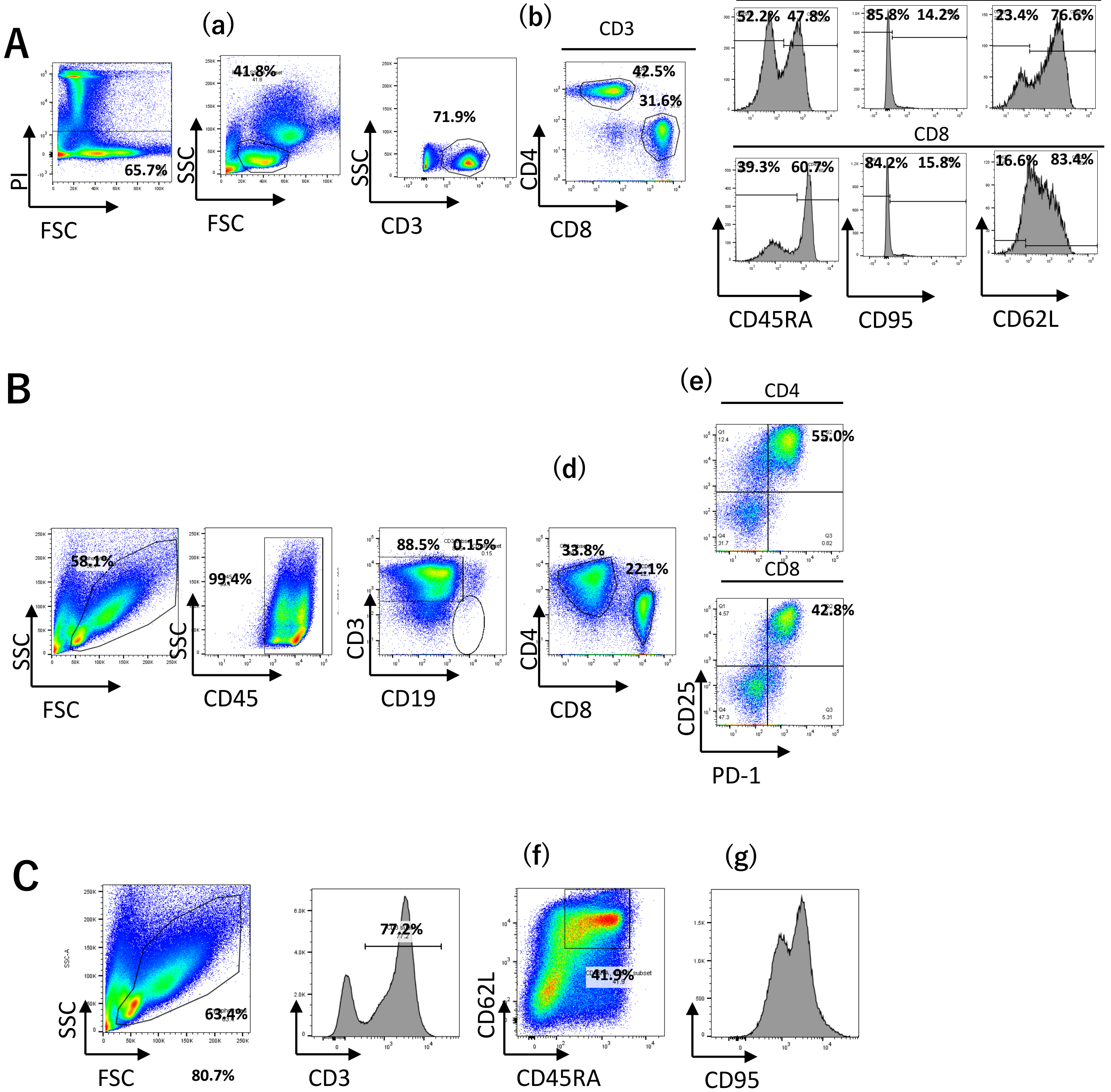

Figure S1. Typical patterns of naïve human PBMC analysis using flow cytometry (FCM) analysis. (A) Living PBMCs were selected as propidium iodide (PI)-negative cells, and the lymphoid-gated cells were selected using the forward and side scatters (FSC/SSC) parameters (a). CD3-gated cells were further divided based on CD4 and CD8 expression (b). Each cell fraction was further analyzed for CD45RA, CD95, and CD62L expression (c). (B) TSST-1-activated T cell analysis. Lymphoid-gated cells were further gated for human CD45 expression. The CD3+ T cells were further gated for CD4 and CD8 expressions (d). Each fraction was further analyzed for CD25 and PD-1 expression (e). (C) Memory T cell analysis. Lymphoid-gated cells were further gated for CD3+ T cells. The CD62L and CD45RA –double-positive cells (f) were further analyzed for CD95 expression (g). The CD95+ cells were presumed to be stem cell like-memory T cells, and CD95- cells are naïve T cells.

Figure S2

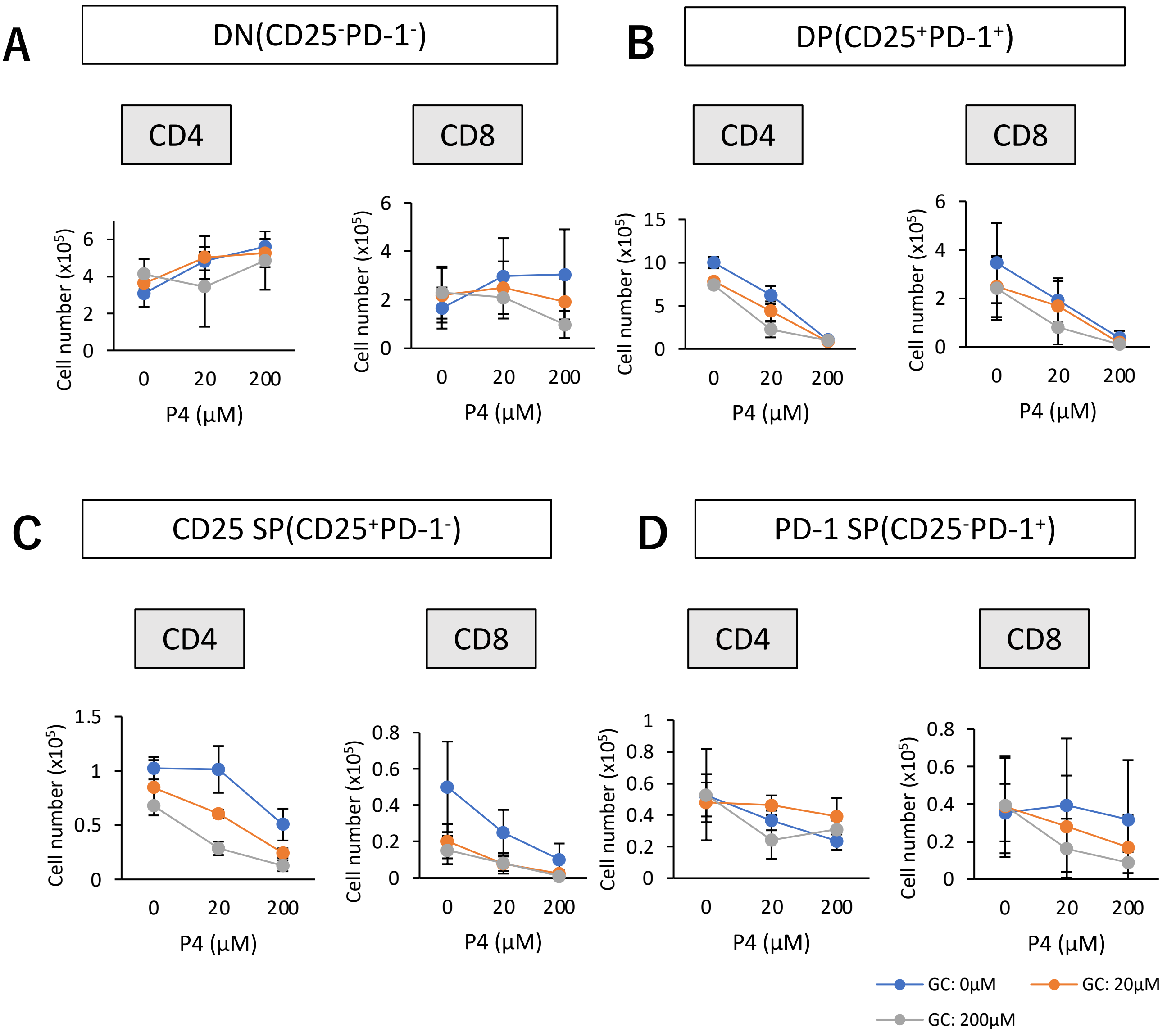
A
DN(CD25⁻PD-1⁻)
B
DP(CD25⁺PD-1⁺)
C
CD25 SP(CD25⁺PD-1⁻)
D
PD-1 SP(CD25⁻PD-1⁺)
CD4
CD8
Cell number (x10^5)
P4 (μM)
0
20
200
GC: 0μM
GC: 20μM
GC: 200μM

Figure S2. The cell numbers of cultured PBMCs. (A) CD25/PD-1 double negative (DN) cells (B) double-positive (DP) cells, (C) CD25 single-positive (CD25 SP) cells (D) PD-1 single positive (PD-1 SP) cells. Blue lines; no other steroids were added. Orange lines, 20 μM COR for the P4 kinetics and P4 for the COR kinetics were added; gray lines, 200 μM COR for the P4 kinetics and P4 for the COR kinetics were added; Left panels of each analysis, CD4 T cells in CD3 T cells; Right panels of each analysis, CD8 T cells in CD3 T cells.

Figure S3

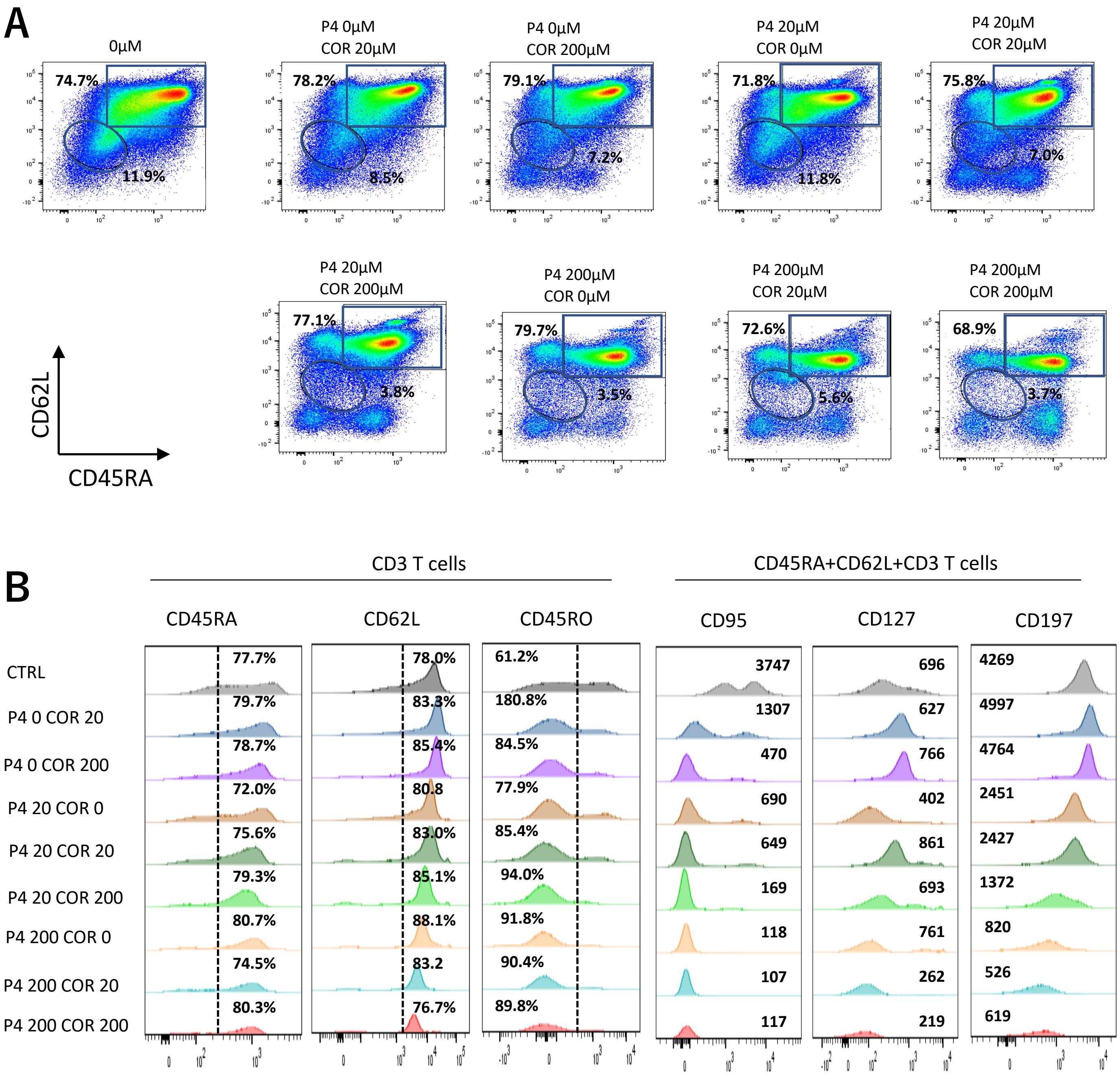

Figure S3. Expression of the differentiation markers on activated T cells in the presence of P4/COR. (A) Typical FCM patterns of CD45RA and CD62L expression on the CD3 T cells cultured for 72 h in the presence of P4 and/or COR. Lymphocyte-gated CD3+ cells analyzed for the expression of CD45RA (vertical axis) and CD62L (horizontal axis). Open squares show the gate of DP cells. The percentages of DP cells are shown in the panels. (B) Left 3 panels: The expression of CD45RA, CD62L, and CD45RO in CD3+ P4/COR treated T cells; Right 3 panels: The expression of CD95, CD127, and CD197 in the CD3+CD45RA+CD62L+ P4/COR treated T cells.

Figure S4

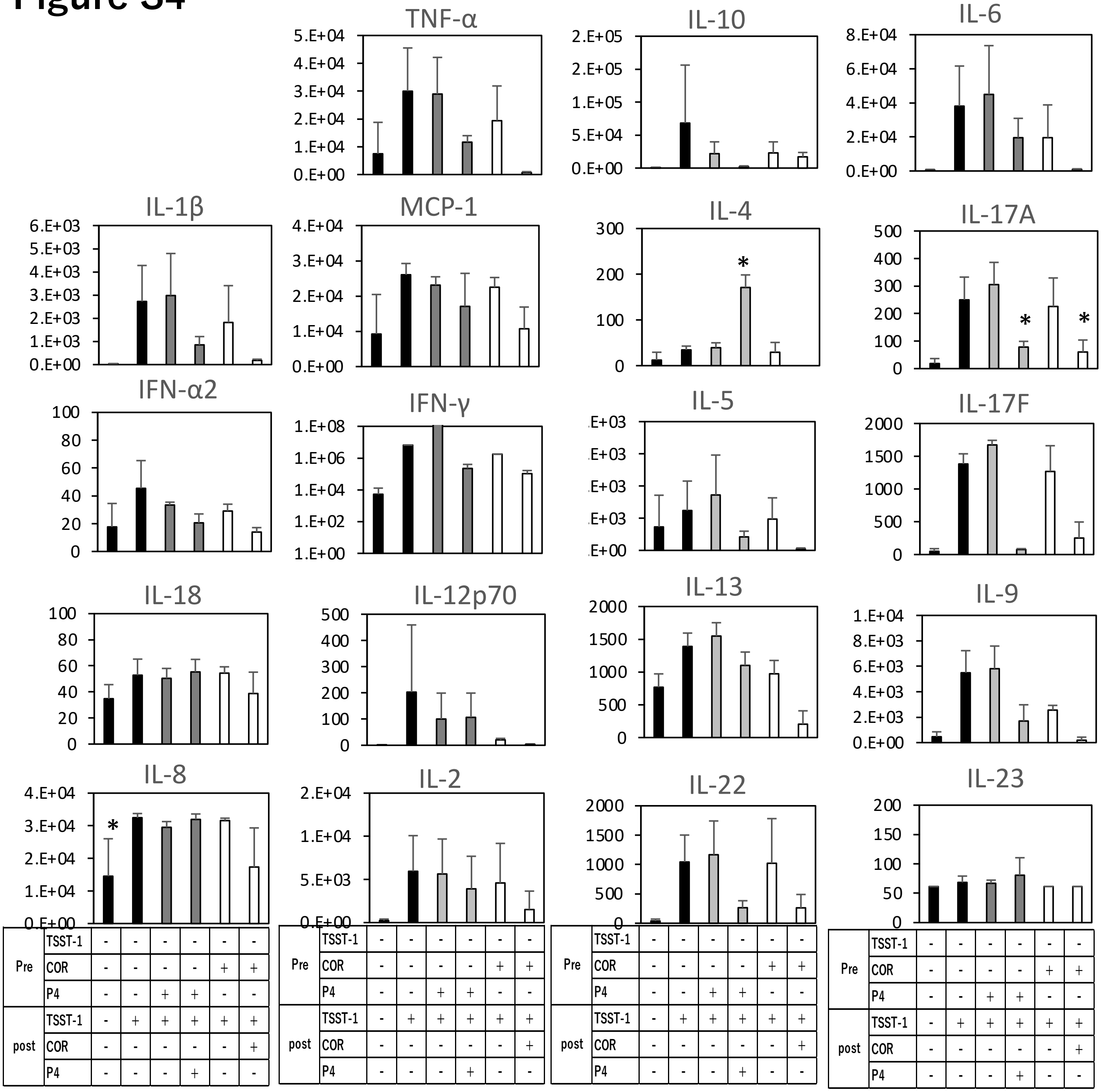

Pre	TSST-1	-	-	-	-	-	-
	COR	-	-	-	-	+	+
	P4	-	-	+	+	-	-
post	TSST-1	-	+	+	+	+	+
	COR	-	-	-	-	-	+
	P4	-	-	-	+	-	-

Pre	TSST-1	-	-	-	-	-	-
	COR	-	-	-	-	+	+
	P4	-	-	+	+	-	-
post	TSST-1	-	+	+	+	+	+
	COR	-	-	-	-	-	+
	P4	-	-	-	+	-	-

Pre	TSST-1	-	-	-	-	-	-
	COR	-	-	-	-	+	+
	P4	-	-	+	+	-	-
post	TSST-1	-	+	+	+	+	+
	COR	-	-	-	-	-	+
	P4	-	-	-	+	-	-

Pre	TSST-1	-	-	-	-	-	-
	COR	-	-	-	-	+	+
	P4	-	-	+	+	-	-
post	TSST-1	-	+	+	+	+	+
	COR	-	-	-	-	-	+
	P4	-	-	-	+	-	-

Figure S4. Concentration of inflammatory and T cell subset-related cytokines in the culture supernatants of P4 pre-treatment and P4 treatment (pre + post), COR pre-treatment, and COR treatment (pre + post) groups. Negative control and positive control are also shown. Vertical axis; cytokine concentration (pg/mL). The horizontal axis of each treatment is shown in the table below. Each data are average ± SD; n = 3; * $p<0.05$ (One way ANOVA).

Figure S5

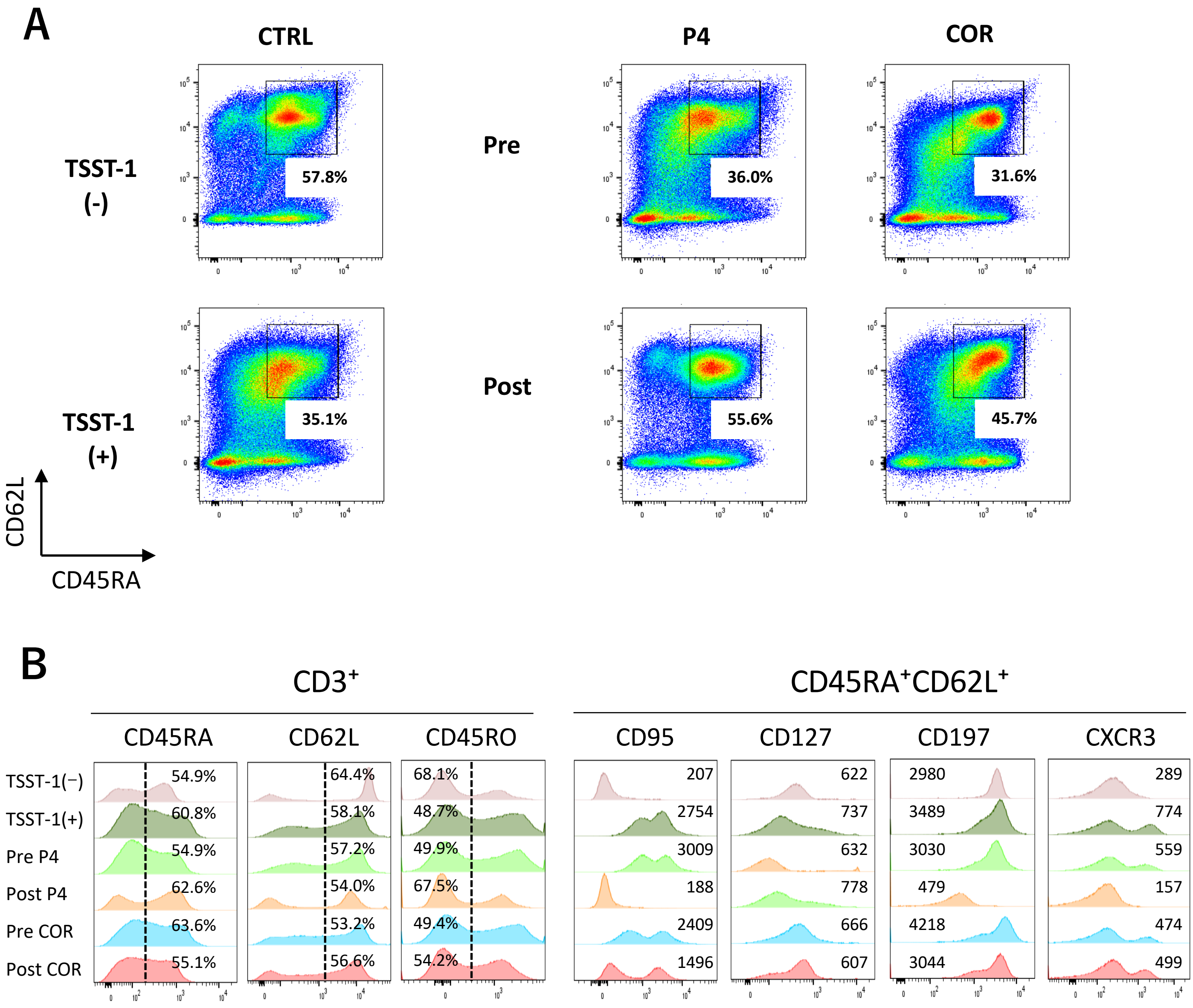

Figure S5. Expression of the differentiation markers on activated T cells of P4 pre-treatment and P4 treatment (pre + post), COR pre-treatment, and COR treatment (pre + post) groups. (A) Typical FCM patterns of CD45RA and CD62L expression on the CD3 T cells of negative control (TSST-1(−)), positive control (TSST-1 (+)), COR pre-treatment group (Pre COR), COR treatment group (Post COR)), P4 pre-treatment group (Pre P4) and P4 treatment group (Post P4). Lymphocyte-gated CD3+ cells were analyzed for the expression of CD45RA (vertical axis) and CD62L (horizontal axis). Open squares show the gate of DP cells. The percentages of DP cells are shown in the panels. (B) Left 3 panels, the expression of CD45RA, CD62L, and CD45RO in $CD3^+$ P4/COR treated T cells; Right 3 panels, the expression of CD95, CD127, and CD197 in the $CD3^+CD45RA^+CD62L^+$ P4/COR treated T cells.

Figure S6

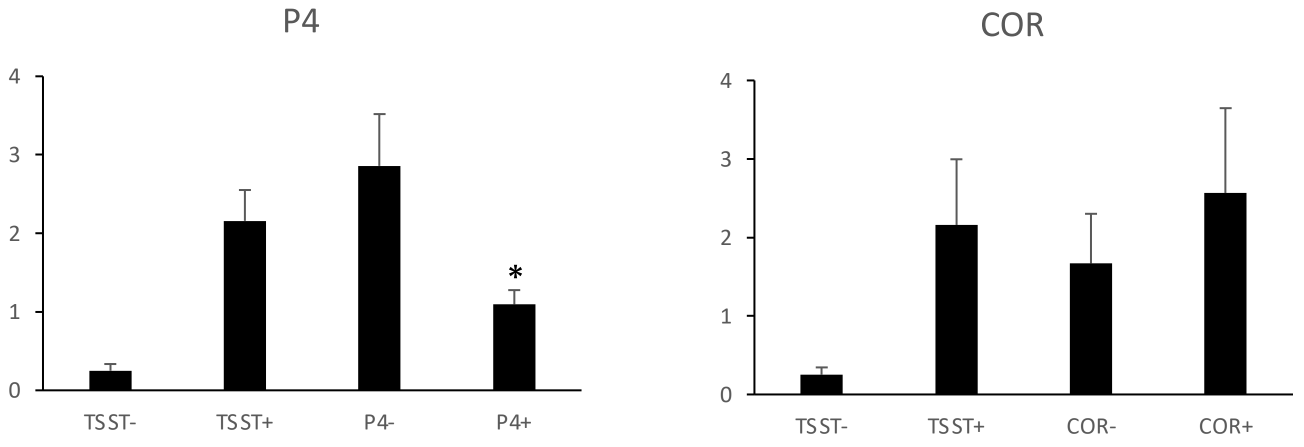

Figure S6. Real-time PCR (RT-PCR) of FOXM1 (a member of the Forkhead Box (Fox) family of transcription factors). P4−; P4 pre-treatment, P4+; P4 pre and post-treatment. COR−; COR pre-treatment, COR+; COR pre and post-treatment. Vertical axis; relative expression level of FOXM1. Two experiments were conducted, and the results are individually shown.

Figure S7

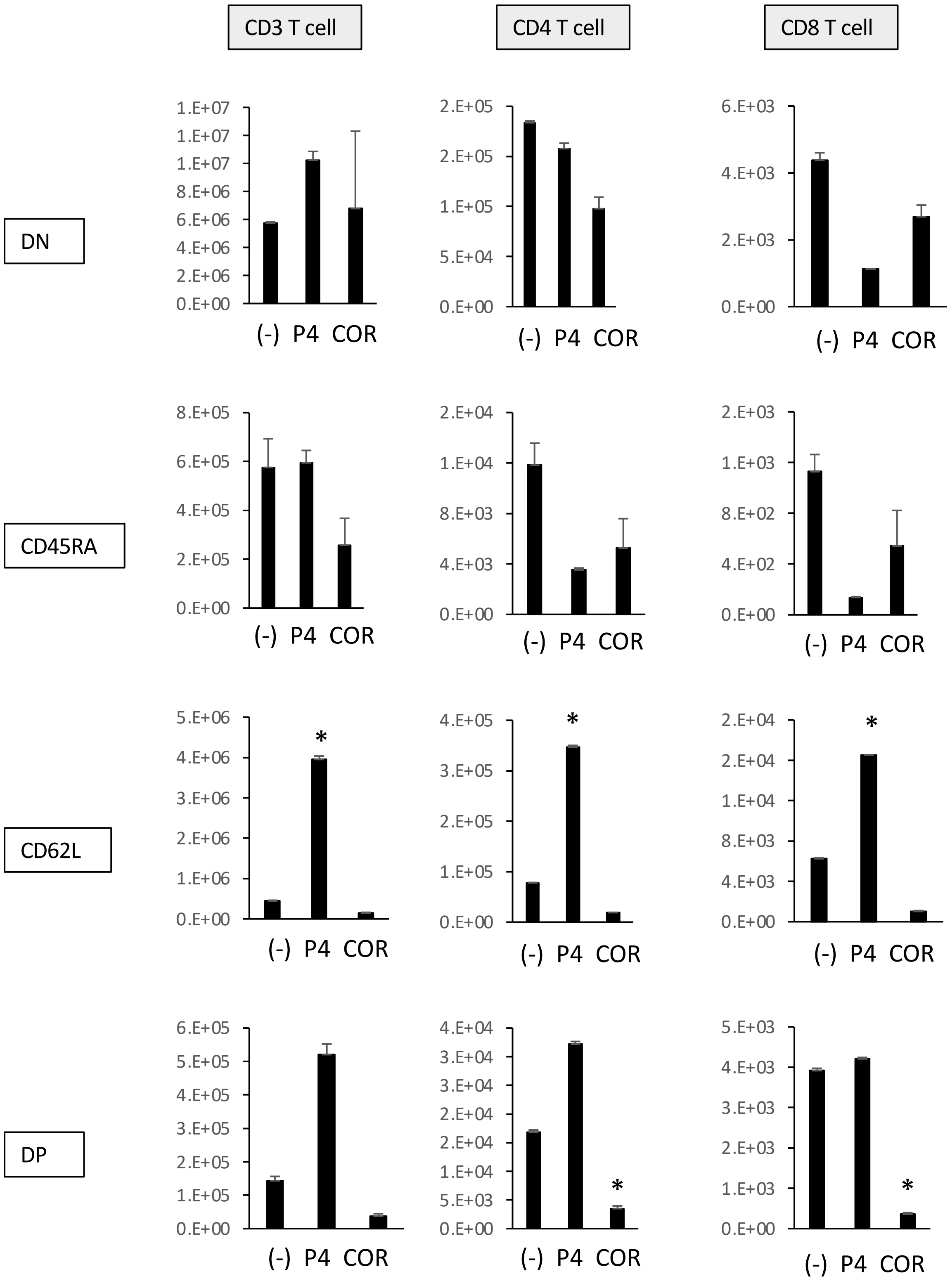

Figure S7. The cell numbers engrafted in humanized mice. Upper panels; CD45RA/CD62L DN and DP cells, Lower panels; CD25 SP and PD-1 SP cells. Blue lines; no other steroids were added. Orange lines; 20 μM COR for the P4 kinetics and P4 for the COR kinetics were added, gray lines; 200 μM COR for the P4 kinetics and P4 for the COR kinetics were added. Left panels of each analysis; CD4 T cells in CD3 T cells, Right panels of each analysis; CD8 T cells in CD3 T cells.
